# Global, regional and national mortality burden of laryngeal cancer attributable to occupational exposure to sulfuric acid and asbestos: 1990–2021 and projections to 2040

**DOI:** 10.3389/fpubh.2025.1602789

**Published:** 2025-07-18

**Authors:** Xin Gong, Yinxia Xu, Yong Hao, Meitao Yi, Chao Yuan, Zhenhong Zheng, Jia Gong, Gongjie Shen, Yongqi Dong

**Affiliations:** ^1^Department of Otolaryngology, Wushan County People's Hospital of Chongqing, Wushan, Chongqing, China; ^2^Children Health Care Center, Wushan County People's Hospital of Chongqing, Wushan, Chongqing, China; ^3^Department of Gastroenterology, Wushan County People's Hospital of Chongqing, Wushan, Chongqing, China

**Keywords:** laryngeal cancer, occupational exposure, sulfuric acid, asbestos, mortality, global burden of disease

## Abstract

**Background:**

Laryngeal cancer (LC) is the most prevalent form of head and neck cancer, significantly impacting patients' health. Occupational exposure to sulfuric acid (OESA) and asbestos (OEA) is a recognized risk factor for LC, but the associated mortality burden remains unclear. This study aimed to evaluate the global trends of LC attributable to OESA and OEA, and to project future trends.

**Methods:**

Based on the Global Burden of Disease Study 2021, we analyzed the number of deaths, age-standardized death rates (ASDR), and estimated annual percentage changes by age, sex, and socio-demographic index (SDI) of LC attributed to OESA and OEA. Decomposition analysis was used to identify the drivers of disease burden changes. Frontier analysis was used to estimate achievable outcomes based on development levels. Additionally, Bayesian age-period-cohort model was used to predict future trends up to 2040.

**Results:**

In 2021, global LC deaths attributable to OESA and OEA were 3,612.35 (95% uncertainty intervals (UI): 1,504.31–6,492.29) and 3,392.00 (95% UI: 1,892.13–5,134.88), respectively, representing increases of 37.5% and 21.4%, respectively, from 1990. The ASDRs for OESA and OEA in 2021 were both 0.04 per 100,000 (95% UI: 0.02–0.07 and 0.02–0.06, respectively), both lower than in 1990. LC deaths attributable to OESA and OEA mainly occur in elderly patients, and men consistently showed higher LC deaths and ASDR than females. The ASDR for OESA was negatively correlated with the SDI, while OEA was positively correlated. Decomposition analysis highlighted differences in disease burden drivers across SDI regions. Frontier analysis showed that countries like Cuba and Pakistan were farthest from the OESA-related mortality burden frontier, while Monaco and Lesotho were farthest from OEA-related mortality burden. From 2022 to 2040, LC deaths attributable to OESA and OEA are projected to increase to 4,810.90 (95% UI: 1,628.04–8,010.74) and 3,648.48 (95% UI: 1,016.75–6,322.42), respectively.

**Conclusions:**

Although the ASDR decreased from 1990 to 2021, OESA and OEA remain contributors to LC mortality worldwide, with the number of deaths expected to increase over the next two decades. Disease burden varies significantly across SDI regions, suggesting that preventive measures should be tailored to specific SDI levels.

## 1 Introduction

Laryngeal cancer (LC) is one of the most prevalent malignant tumors of the head and neck (1). In 2021, ~200,883 new cases and 117,252 deaths from LC were reported worldwide, with age-standardized incidence and mortality rates of 2.293 and 1.35 per 100,000, respectively (2). The incidence and mortality rates of LC are approximately five times higher in men than in females. Most patients are diagnosed at an advanced stage, except for patients with early glottic cancer who seek early treatment due to hoarseness (3). Despite medical advances, patient survival rates and quality of life remain suboptimal (4). The occurrence and progression of LC significantly impairs vocalization and swallowing functions, posing a significant physical and psychological burden, particularly in low- and middle-income countries with limited access to healthcare (1, 2). For example, the Caribbean had the highest incidence (ASIR = 4.0) and mortality (ASMR = 2.1) rate of LC in 2021 (1).

As industrialization progresses, a growing number of carcinogens have been identified significant contributors to global, regional, and national disease burden (5–7). Sulfuric acid mist and asbestos are among the most common occupational carcinogens and are classified as Group 1 carcinogens by the International Agency for Research on Cancer (8). Sulfuric acid, a highly corrosive inorganic acid, is widely used in paper manufacturing, detergent production, explosive formulation, and fertilizer synthesis (9). Asbestos is a group of silicate mineral products that are widely used in roofing materials, textiles, friction materials, and cement pipes (10). Industrial products derived from sulfuric acid and asbestos are integral to daily life, making exposure nearly unavoidable. Numerous epidemiological and cohort studies have confirmed that exposure to sulfuric acid mist is associated with an elevated risk of developing respiratory tract cancers (9, 10). According to the GBD 2019 study, ~239,330 deaths and 4,189,000 disability-adjusted life years (DALYs) worldwide due to occupational asbestos exposure occurred in 2019 (7). The carcinogenic effects of asbestos have been widely recognized, and while mesothelioma is the primary malignant neoplasm associated with asbestos exposure (7, 11). Numerous studies have demonstrated that occupational exposure to sulfuric acid (OESA) and asbestos (OEA) is significantly related to an increased risk of developing LC (7, 9–13).

Exploring the epidemiological relationships between preventable risk factors and LC is essential for the development of effective prevention and treatment policies. While previous studies have assessed the epidemiological trends of LC (1, 2, 14), they lack detailed analyses of the global mortality burden attributable to various risk factors. Occupational hazards have received considerable attention, prompting many countries to implement policies aimed at reducing the harm caused by substances such as sulfuric acid and asbestos (15–17). However, the effectiveness of these policies in reducing LC burden remains unclear. Utilizing the Global Burden of Disease (GBD) 2021 study, we provide a comprehensive analysis of the mortality burden of LC attributable to OESA and OEA at the global, regional, and national levels, along with trends from 1990 to 2021, and project future trends up to 2040. These findings are expected to inform evidence-based strategies for the prevention of LC attributable to OESA and OEA.

## 2 Methods

### 2.1 Overview

The GBD study provides a comprehensive assessment of the epidemiological levels and trends associated with communicable diseases, noncommunicable diseases, and injuries worldwide. This study utilized data retrieved from the GBD 2021, which systematically evaluated 371 diseases and 88 risk factors across 204 countries and territories (18). For GBD 2021, data aggregation entailed a comprehensive collection from various sources, including population censuses, household surveys, civil registration records, disease registries, health service use data, air quality monitoring, satellite imagery, and other related health databases. DisMod-MR 2.1, a Bayesian meta-regression tool developed for the GBD study, is designed to estimate non-fatal health outcomes using sparse and heterogeneous epidemiological data and provides a comprehensive view of diseases. Further information can be accessed on the Institute for Health Metrics and Evaluation (IHME) website (https://vizhub.healthdata.org/gbd-results/).

### 2.2 Definition and data sources

In the GBD database, LC is classified under the International Statistical Classification of Diseases and Related Health Problems 10th Revision code C32, which includes several subcategories: C32.0, glottis; C32.1, supraglottis; C32.3, laryngeal cartilage; C32.8, overlapping lesions of the larynx; C32.9, unspecified locations within the larynx. In our data search, we used “laryngeal cancer” as the Cause, while “Occupational exposure to sulfuric acid” and “Occupational exposure to asbestos” served as the two risk factors under investigation. We measured ‘deaths' and age-standardized death rate (ASDR) from 1990 to 2021, utilizing metrics such as number, percent, and rate. Data on the number of deaths and ASDR, along with their 95% uncertainty intervals (UI), were collected. These data were further analyzed by sex, age, region, and country. We categorized the world into 21 regions based on epidemiological similarities and geographic proximity. Age distribution was analyzed using 16 age categories based on 4-year age group intervals to investigate patterns in mortality by age.

The socio-demographic index (SDI), a comprehensive measure introduced by the IHME in 2015, evaluates the social and economic circumstances that influence health outcomes across countries and regions at varying levels of development. In the GBD 2021 study, 204 countries and territories were classified into five development levels based on their SDI values: low ( ≤ 0.4658), low-middle (0.4658–0.6188), middle (0.6188–0.7120), high-middle (0.7120–0.8103), and high (>0.8103) (19).

### 2.3 Statistical analysis

Age-standardized rates (ASR) are calculated based on the world standard population developed by the GBD study via the following formula (18). ASDR was used to evaluate and compare death rates across nations or regions with varying age structures and demographic characteristics. The estimated annual percentage change (EAPC) serves as a commonly adopted metric for characterizing the trends of ASR across specific time intervals. The EAPC was calculated to gain a deeper understanding of the temporal trends in LC death burden. A regression line was fitted to the natural logarithm of the ratio, using the equation:


$$\begin{array}{l} \text{y}=\alpha +\beta \text{x}+\text{e}, \end{array}$$

where y = ln(ASDR) and x = calendar year. With this linear regression equation, the EAPC was calculated using the equation:


$$\begin{array}{l} \text{EAPC}=100\times \left(\text{exp}\left[\beta \right]-\;1\right), \end{array}$$

with a 95% confidence interval (CI), where β denotes the slope of the log-linear regression model (20). A positive EAPC indicates an increasing trend, while a negative EAPC indicates a declining trend. If the 95% CI of the EAPC estimate includes 0, the ASDR is relatively stable.

### 2.4 Decomposition analysis

Decomposition analysis fundamentally involves dissecting the total variation in an outcome into the individual contributions of different factors (21). We used decomposition analysis to evaluate changes in LC deaths attributable to aging, population growth, and epidemiologic changes. This approach allowed us to quantify the independent contributions of each factor to the overall mortality trends, aiding in the identification of priority areas for intervention.

### 2.5 Frontier analysis

Considering the socio-demographic development of various regions, frontier analysis (22) was used to explore the potential for reducing the disease burden. This analysis established a frontier for the lowest achievable ASDR of LC at each SDI level, identifying regions where LC health outcomes lag behind developmental expectations to reveal improvement opportunities and guide strategies for optimizing outcomes related to OESA and OEA.

### 2.6 Bayesian age-period-cohort (BAPC) model

The BAPC model, a Bayesian statistical methodology, discerns the distinct influences of age, period, and cohort on health outcomes (23). It melds prior and sample data within the Bayesian generalized linear model paradigm, leveraging a second-order random walk to smooth effects and generate accurate posterior probability forecasts. The Integrated Nested Laplace Approximation (INLA) technique is integral to the BAPC model. It elevates computational efficiency and enables model comparison via the Deviance Information Criterion (DIC), sidestepping the mixing and convergence issues typical of Markov Chain Monte Carlo methods. The BAPC model furnishes uncertainty estimates and navigates data intricacies with lower error rates than alternative models, making it a prime choice for investigating long-term disease burden trends. This model was used to project future mortality trends and analyze LC deaths and ASDR from 2022 to 2040.

All analyses were performed using R software (version 4.3.3). Statistical significance was set at *p* < 0.05. Our study was conducted in line with the Guidelines for Accurate and Transparent Health Estimates Reporting (GATHER) (24).

## 3 Results

### 3.1 Global trends

In 2021, estimated global deaths from LC attributable to OESA and OEA were 3,612.35 (95% UI: 1,504.31–6,492.29) and 3,392.00 (95% UI: 1,892.13–5,134.88), respectively. Compared to 1990, deaths increased by 37.5% for OESA (from 2,626.69 [95% UI: 1,047.38–4,839.19]) and by 21.4% for OEA (from 2,794.84 [95% UI: 1,605.96–4,157.60]). The ASDR for LC attributable to OESA decreased by 33% from 0.06 (95% UI: 0.02–0.11) in 1990 to 0.04 (95% UI: 0.02–0.07) in 2021 per 100,000 population. Similarly, the ASDR for LC attributable to OEA decreased by 50% from 0.08 (95% UI: 0.04–0.11) in 1990 to 0.04 (95% UI: 0.02–0.06) in 2021 per 100,000 population. The EAPC of ASDR from 1990 to 2021 was −1.56 (95% CI: −1.64 to −1.49) for OESA and −2.01 (95% CI: −2.09 to −1.93) for OEA (Tables 1, 2).

**Table 1:** The number of deaths and ASDR of LC attributable to OESA in 1990 and 2021, and EAPC from 1990 to 2021 at the global and regional level.

**Location**	**1990**		**2021**		**1990–2021**
	**Number of deaths, (95% UI)**	**ASDR** **per 100 000** **(95% UI)**	**Number of deaths, (95% UI)**	**ASDR** **per 100 000** **(95% UI)**	**EAPC, %, (95% CI)**
Global	2,626.69 (1,047.38, 4,839.19)	0.06 (0.02, 0.11)	3,612.35 (1,504.31, 6,492.29)	0.04 (0.02, 0.07)	−1.56 (−1.64, −1.49)
**SDI**					
High	407.21 (106.42, 867.98)	0.04 (0.01, 0.08)	324.22 (87.26, 664.53)	0.02 (0.00, 0.04)	−2.78 (−2.91, −2.65)
High–middle	786.73 (296.61, 1,499.05)	0.07 (0.03, 0.14)	696.96 (274.69, 1,319.30)	0.03 (0.01, 0.07)	−2.83 (−2.95, −2.70)
Middle	750.29 (306.89, 1,411.86)	0.06 (0.03, 0.12)	1,283.21 (539.81, 2,347.34)	0.04 (0.02, 0.08)	−1.41 (−1.47, −1.35)
Low–middle	520.67 (207.35, 958.86)	0.07 (0.03, 0.14)	1,030.84 (419.84, 1,874.12)	0.07 (0.03, 0.12)	−0.46 (−0.52, −0.40)
Low	158.36 (60.50, 303.20)	0.06 (0.02, 0.11)	272.79 (106.70, 519.58)	0.05 (0.02, 0.09)	−1.02 (−1.13, −0.92)
**Regions**					
Andean Latin America	7.67 (3.13, 14.43)	0.04 (0.01, 0.07)	13.59 (5.48, 25.57)	0.02 (0.01, 0.04)	−1.70 (−1.88, −1.53)
Australasia	6.60 (1.59, 14.73)	0.03 (0.01, 0.06)	4.69 (1.12, 10.54)	0.01 (0.00, 0.02)	−3.62 (−3.77, −3.48)
Caribbean	22.40 (9.02, 42.08)	0.08 (0.03, 0.16)	53.15 (22.72, 101.20)	0.10 (0.04, 0.19)	0.71 (0.59, 0.84)
Central Asia	53.94 (22.08, 98.25)	0.10 (0.04, 0.19)	33.32 (13.53, 62.37)	0.03 (0.01, 0.06)	−3.70 (−3.92, −3.49)
Central Europe	134.94 (31.61, 294.24)	0.09 (0.02, 0.19)	119.70 (27.71, 252.49)	0.06 (0.01, 0.13)	−1.53 (−1.66, −1.41)
Central Latin America	57.14 (24.01, 104.30)	0.07 (0.03, 0.12)	82.17 (33.67, 146.99)	0.03 (0.01, 0.06)	−2.83 (−2.98, −2.69)
Central Sub–Saharan Africa	10.64 (3.79, 20.95)	0.04 (0.01, 0.08)	18.46 (7.03, 35.39)	0.03 (0.01, 0.05)	−1.33 (−1.44, −1.22)
East Asia	528.28 (213.20, 1,027.87)	0.05 (0.02, 0.11)	821.40 (331.63, 1,581.91)	0.03 (0.01, 0.07)	−1.65 (−1.75, −1.56)
Eastern Europe	239.02 (57.35, 523.37)	0.08 (0.02, 0.18)	101.90 (25.32, 227.35)	0.03 (0.01, 0.07)	−3.85 (−4.10, −3.61)
Eastern Sub–Saharan Africa	41.14 (15.93, 78.60)	0.05 (0.02, 0.09)	82.56 (31.39, 159.66)	0.04 (0.02, 0.08)	−0.71 (−0.82, −0.60)
High–income Asia Pacific	41.86 (10.15, 91.84)	0.02 (0.00, 0.04)	26.74 (5.98, 58.20)	0.01 (0.00, 0.01)	−4.03 (−4.19, −3.87)
High–income North America	99.37 (23.59, 220.62)	0.03 (0.01, 0.07)	102.37 (24.59, 222.03)	0.02 (0.00, 0.04)	−2.41 (−2.54, −2.29)
North Africa and Middle East	116.71 (47.90, 221.13)	0.06 (0.02, 0.11)	180.92 (75.66, 336.29)	0.04 (0.01, 0.07)	−1.85 (−1.93, −1.77)
Oceania	0.29 (0.11, 0.59)	0.01 (0.00, 0.02)	0.67 (0.26, 1.31)	0.01 (0.00, 0.01)	−0.33 (−0.38, −0.28)
South Asia	663.77 (265.92, 1,235.79)	0.10 (0.04, 0.18)	1,243.55 (516.03, 2,290.45)	0.08 (0.03, 0.14)	−0.96 (−1.05, −0.87)
Southeast Asia	106.59 (44.60, 196.51)	0.04 (0.02, 0.07)	267.13 (109.94, 496.23)	0.04 (0.01, 0.07)	−0.08 (−0.11, −0.05)
Southern Latin America	56.41 (22.42, 105.83)	0.12 (0.05, 0.22)	37.15 (15.11, 69.00)	0.04 (0.02, 0.08)	−3.06 (−3.15, −2.96)
Southern Sub–Saharan Africa	15.34 (6.21, 28.57)	0.05 (0.02, 0.09)	13.86 (5.32, 26.75)	0.02 (0.01, 0.04)	−3.05 (−3.47, −2.63)
Tropical Latin America	99.46 (40.50, 180.24)	0.10 (0.04, 0.18)	193.98 (79.34, 365.72)	0.07 (0.03, 0.14)	−0.99 (−1.17, −0.81)
Western Europe	299.08 (72.92, 662.02)	0.06 (0.01, 0.13)	164.78 (38.55, 362.48)	0.02 (0.00, 0.05)	−3.34 (−3.48, −3.21)
Western Sub–Saharan Africa	26.03 (10.49, 49.56)	0.03 (0.01, 0.05)	50.23 (20.71, 96.12)	0.02 (0.01, 0.04)	−0.68 (−0.77, −0.60)

LC, laryngeal cancer; ASDR, age–standardized death rate; OESA, occupational exposure to sulfuric acid; EAPC, estimated annual percentage change; SDI, socio–demographic index; UI, uncertainty intervals; CI, confidence intervals.

**Table 2:** The number of deaths and ASDR of LC attributable to OEA in 1990 and 2021, and EAPC from 1990 to 2021 at the global and regional level.

**Location**	**1990**		**2021**		**1990–2021**
	**Number of deaths, (95% UI)**	**ASDR** **per 100 000** **(95% UI)**	**Number of deaths, (95% UI)**	**ASDR** **per 100 000** **(95% UI)**	**EAPC, %, (95% CI)**
Global	2,794.84 (1,605.96, 4,157.60)	0.08 (0.04, 0.11)	3,392.00 (1,892.13, 5,134.88)	0.04 (0.02, 0.06)	−2.01 (−2.09, −1.93)
**SDI**					
High	1,465.99 (863.39, 2,123.13)	0.13 (0.08, 0.19)	1,512.39 (879.50, 2,192.10)	0.06 (0.04, 0.09)	−2.12 (−2.24, −2.00)
High–middle	928.32 (498.99, 1,392.95)	0.10 (0.05, 0.14)	883.49 (491.67, 1,329.86)	0.04 (0.02, 0.07)	−2.51 (−2.62, −2.40)
Middle	230.50 (131.02, 361.42)	0.03 (0.02, 0.04)	528.48 (284.86, 820.64)	0.02 (0.01, 0.03)	−0.81 (−0.97, −0.65)
Low–middle	128.27 (61.71, 232.14)	0.02 (0.01, 0.04)	370.95 (195.76, 618.17)	0.03 (0.02, 0.05)	0.62 (0.52, 0.72)
Low	38.81 (13.82, 79.93)	0.02 (0.01, 0.04)	92.30 (38.80, 165.03)	0.02 (0.01, 0.04)	0.31 (0.14, 0.49)
**Regions**					
Andean Latin America	6.16 (3.34, 9.72)	0.04 (0.02, 0.06)	8.96 (4.48, 15.51)	0.02 (0.01, 0.03)	−3.04 (−3.55, −2.53)
Australasia	50.21 (29.47, 71.28)	0.21 (0.12, 0.29)	47.70 (28.92, 69.31)	0.08 (0.05, 0.12)	−3.24 (−3.39, −3.10)
Caribbean	11.38 (6.04, 17.57)	0.05 (0.02, 0.07)	20.40 (9.85, 34.16)	0.04 (0.02, 0.06)	−0.75 (−0.88, −0.62)
Central Asia	15.96 (8.89, 24.09)	0.03 (0.02, 0.05)	17.09 (8.82, 27.80)	0.02 (0.01, 0.04)	−1.70 (−1.97, −1.43)
Central Europe	80.55 (42.81, 129.28)	0.05 (0.03, 0.08)	164.38 (90.46, 255.94)	0.07 (0.04, 0.11)	1.69 (1.42, 1.96)
Central Latin America	26.51 (15.00, 38.78)	0.04 (0.02, 0.05)	48.13 (25.03, 75.72)	0.02 (0.01, 0.03)	−1.92 (−2.08, −1.75)
Central Sub–Saharan Africa	2.88 (0.48, 8.39)	0.02 (0.00, 0.04)	6.44 (1.19, 18.60)	0.01 (0.00, 0.04)	−0.22 (−0.55, 0.12)
East Asia	106.72 (58.33, 170.76)	0.02 (0.01, 0.03)	240.19 (128.09, 387.45)	0.01 (0.01, 0.02)	−0.43 (−0.74, −0.11)
Eastern Europe	168.15 (88.74, 256.30)	0.06 (0.03, 0.09)	134.62 (70.72, 215.25)	0.04 (0.02, 0.06)	−2.32 (−2.70, −1.94)
Eastern Sub–Saharan Africa	8.68 (1.16, 25.67)	0.01 (0.00, 0.04)	18.06 (3.27, 52.30)	0.01 (0.00, 0.04)	−0.28 (−0.36, −0.19)
High–income Asia Pacific	62.09 (33.69, 94.31)	0.03 (0.02, 0.05)	134.93 (71.21, 203.23)	0.02 (0.01, 0.03)	−1.15 (−1.35, −0.96)
High–income North America	420.84 (241.39, 609.93)	0.11 (0.06, 0.16)	440.13 (252.43, 636.81)	0.06 (0.04, 0.09)	−2.34 (−2.52, −2.15)
North Africa and Middle East	111.41 (54.16, 190.97)	0.08 (0.04, 0.13)	135.41 (65.12, 229.27)	0.04 (0.02, 0.06)	−2.73 (−3.03, −2.42)
Oceania	0.13 (0.05, 0.24)	0.01 (0.00, 0.01)	0.33 (0.13, 0.61)	0.01 (0.00, 0.01)	0.37 (0.15, 0.59)
South Asia	149.34 (71.42, 275.60)	0.03 (0.01, 0.06)	442.21 (222.86, 764.82)	0.03 (0.02, 0.06)	0.14 (−0.04, 0.32)
Southeast Asia	25.32 (12.91, 42.54)	0.01 (0.01, 0.02)	69.72 (34.13, 117.26)	0.01 (0.01, 0.02)	−0.15 (−0.24, −0.05)
Southern Latin America	38.91 (21.01, 60.58)	0.09 (0.05, 0.13)	55.68 (30.34, 83.26)	0.06 (0.03, 0.09)	−0.38 (−0.76, −0.00)
Southern Sub–Saharan Africa	25.84 (12.67, 44.11)	0.10 (0.05, 0.18)	55.78 (29.72, 89.93)	0.11 (0.06, 0.17)	−0.44 (−1.29, 0.42)
Tropical Latin America	63.37 (36.26, 94.82)	0.08 (0.05, 0.12)	153.03 (85.61, 231.15)	0.06 (0.03, 0.09)	−0.47 (−0.62, −0.31)
Western Europe	1,407.76 (826.25, 2,012.62)	0.23 (0.14, 0.33)	1,178.05 (700.44, 1,673.87)	0.11 (0.07, 0.16)	−2.12 (−2.31, −1.93)
Western Sub–Saharan Africa	12.65 (4.64, 23.95)	0.01 (0.01, 0.03)	20.76 (8.96, 39.62)	0.01 (0.01, 0.02)	−0.72 (−0.82, −0.63)

LC, laryngeal cancer; ASDR, age–standardized death rate; OEA, occupational exposure to asbestos; EAPC, estimated annual percentage change; SDI, socio–demographic index; UI, uncertainty intervals; CI, confidence intervals.

**Figure 1:**
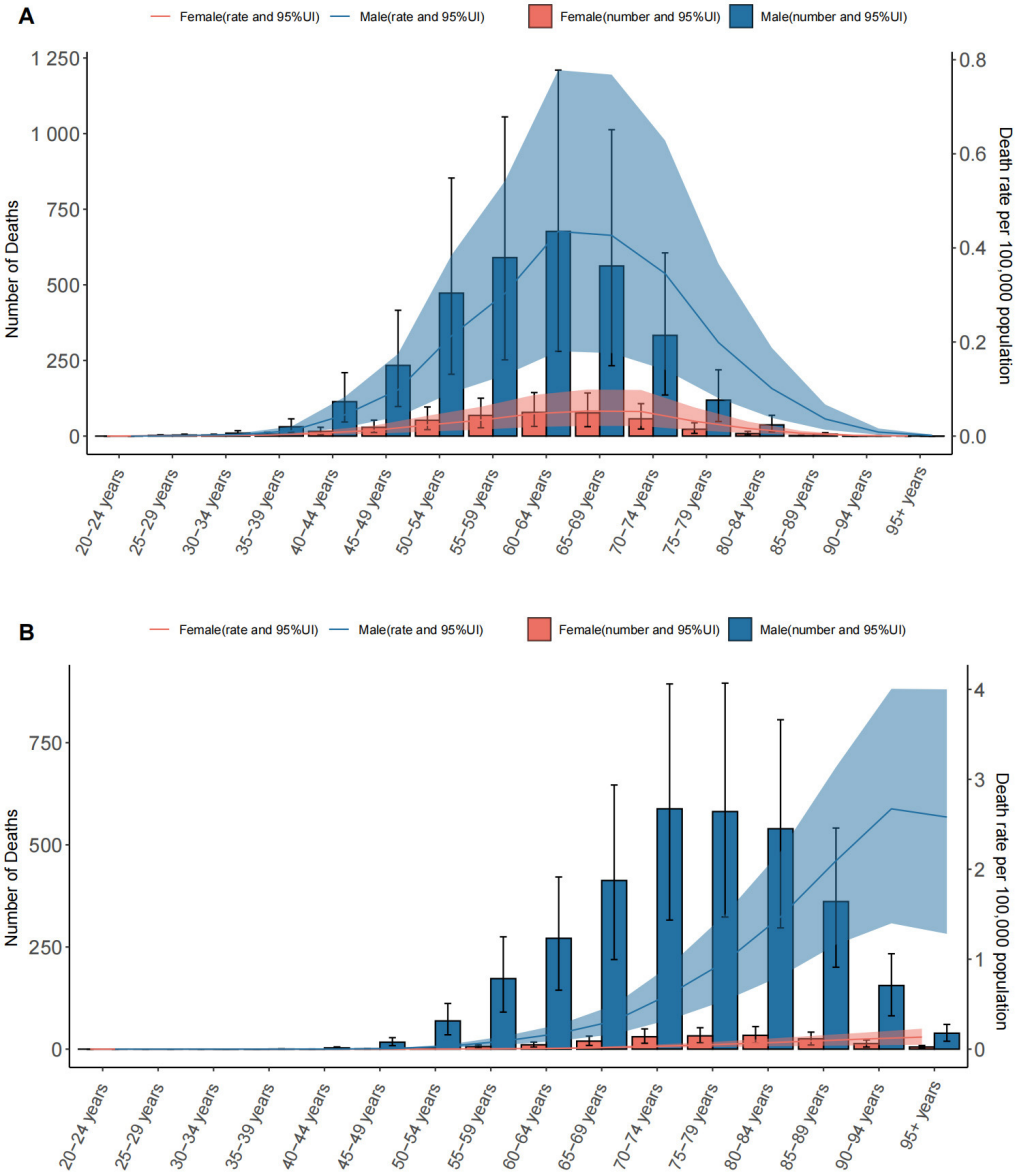
The number of deaths and death rates of LC attributable to OESA **(A)** and OEA **(B)** per 100,000 population by age and sex, in 2021. Error bars indicate the 95% UI for death. Shading indicates the upper and lower limits of the 95% UI. LC, laryngeal cancer; OESA, occupational exposure to sulfuric acid; OEA, occupational exposure to asbestos; UI, uncertainty intervals.

Both exposures resulted in sex- and age-related disparities. LC deaths attributable to OESA and OEA mainly occur in elderly patients. In 2021, The number of deaths also increased with age, showing a clear upward trend starting from the 40–44 age group for OESA, peaking at 60–64 years for both sexes, before declining in older age groups. For OEA, LC deaths exhibited a distinct upward trend beginning at 50–54 years, peaking at 70–74 years for males and 80–84 years for females. ASDR was significantly higher in males and increased with age, peaking in males aged 60–64 years and females aged 65–69 years for OESA. For OEA, peak ASDR occurred in males aged 90–94 years and females aged ≥ 95 years (Figure 1).

### 3.2 Trends by SDI and geographic regions

**Figure 2:**
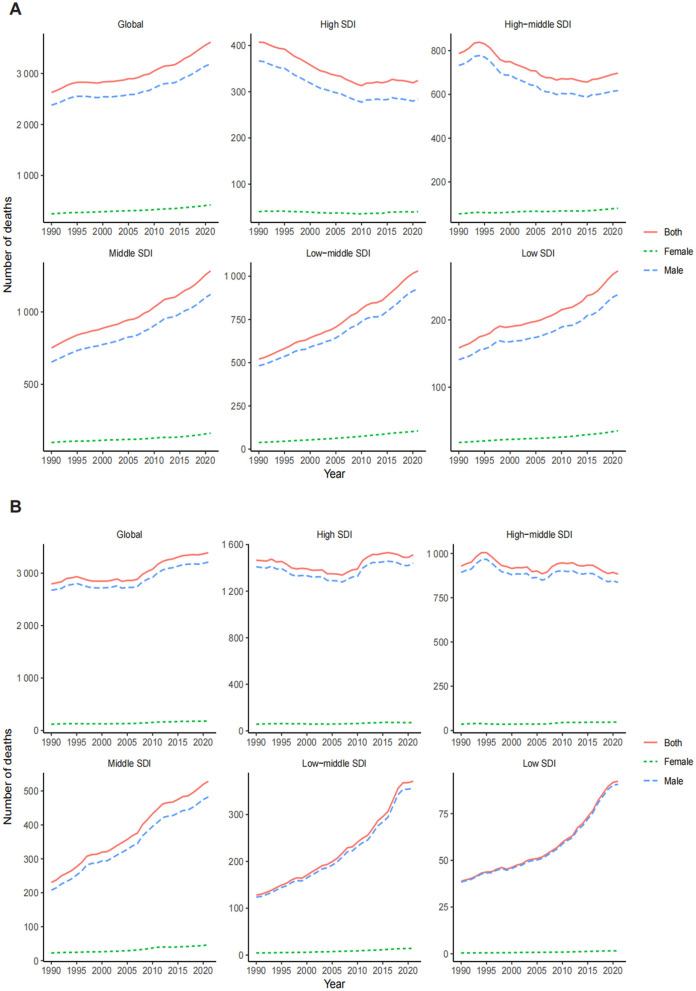
Global trends in the number of deaths for LC attributable to OESA **(A)** and OEA **(B)** from 1990 to 2021 by year, sex, and SDI. LC, laryngeal cancer; OESA, occupational exposure to sulfuric acid; OEA, occupational exposure to asbestos; SDI, socio-demographic index.

The LC death burden attributable to OESA and OEA showed distinct trends regionally. In 1990, the highest number of OESA-related deaths occurred in high-middle SDI regions (786.73, 95% UI: 296.61–1,499.05), shifting to middle SDI regions in 2021 (1,283.21, 95% UI: 539.81–2,347.34). Notably, deaths declined in high-middle and high SDI regions but increased in other SDI regions, especially in low-middle SDI regions, nearly doubling from 520.67 (95% UI: 207.35–958.86) in 1990 to 1,030.84 (95% UI: 419.84–1,874.12) in 2021 (Figure 2A and Table 1). For OEA, high SDI regions had the highest number of deaths in both 1990 (1,465.99, 95% UI: 863.39–2,123.13) and 2021 (1,512.39, 95% UI: 879.50–2,192.10). The high-middle SDI regions experienced a 4.8% decline, while other regions had experienced increases, with the greatest increase in the low-middle SDI regions (Figure 2B and Table 2).

**Figure 3:**
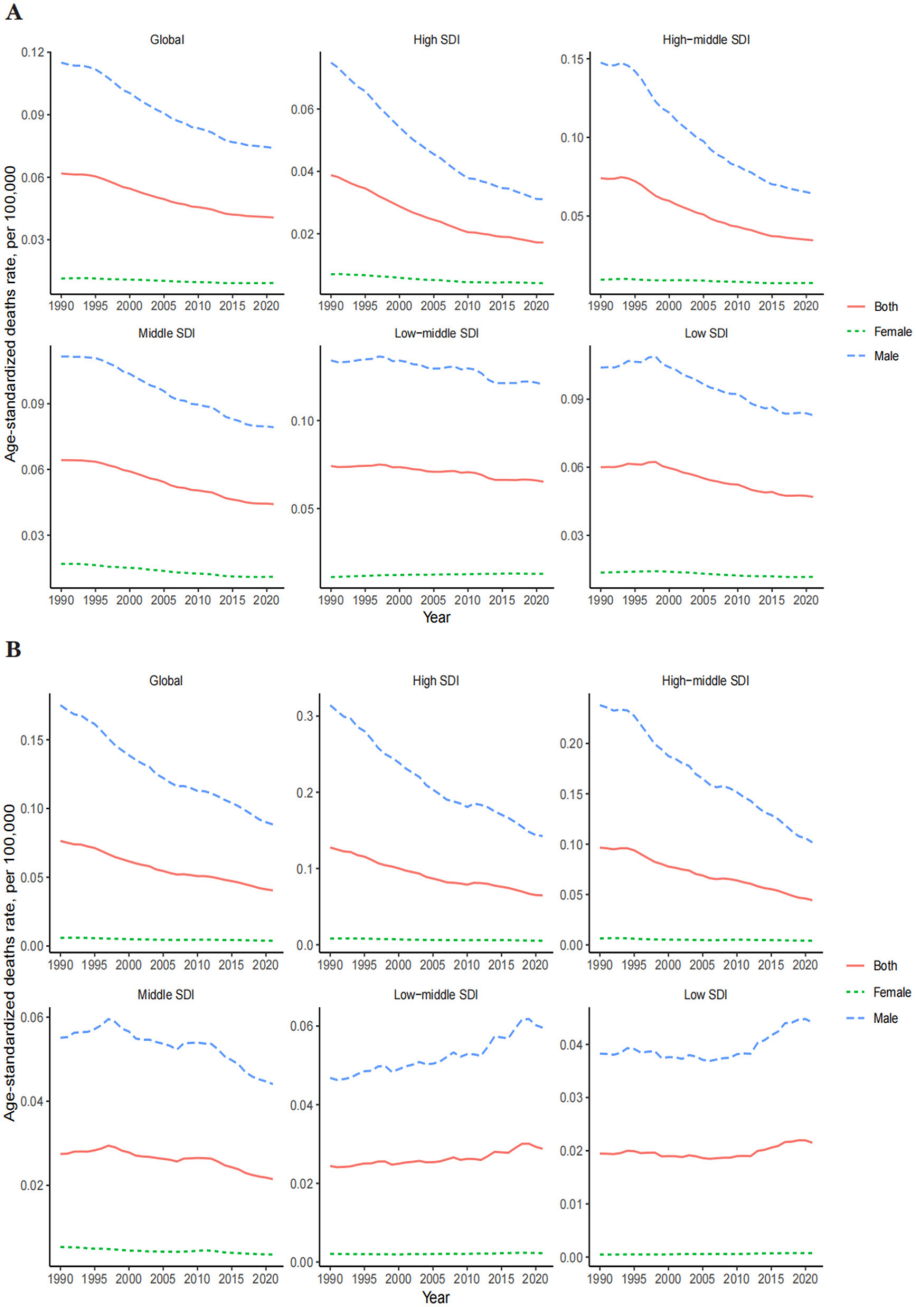
Global trends in the ASDR of LC attributable to OESA **(A)** and OEA **(B)** from 1990 to 2021 by year, sex, SDI. ASDR, age-standardized death rate; LC, laryngeal cancer; OESA, occupational exposure to sulfuric acid; OEA, occupational exposure to asbestos; SDI, socio-demographic index.

**Figure 4:**
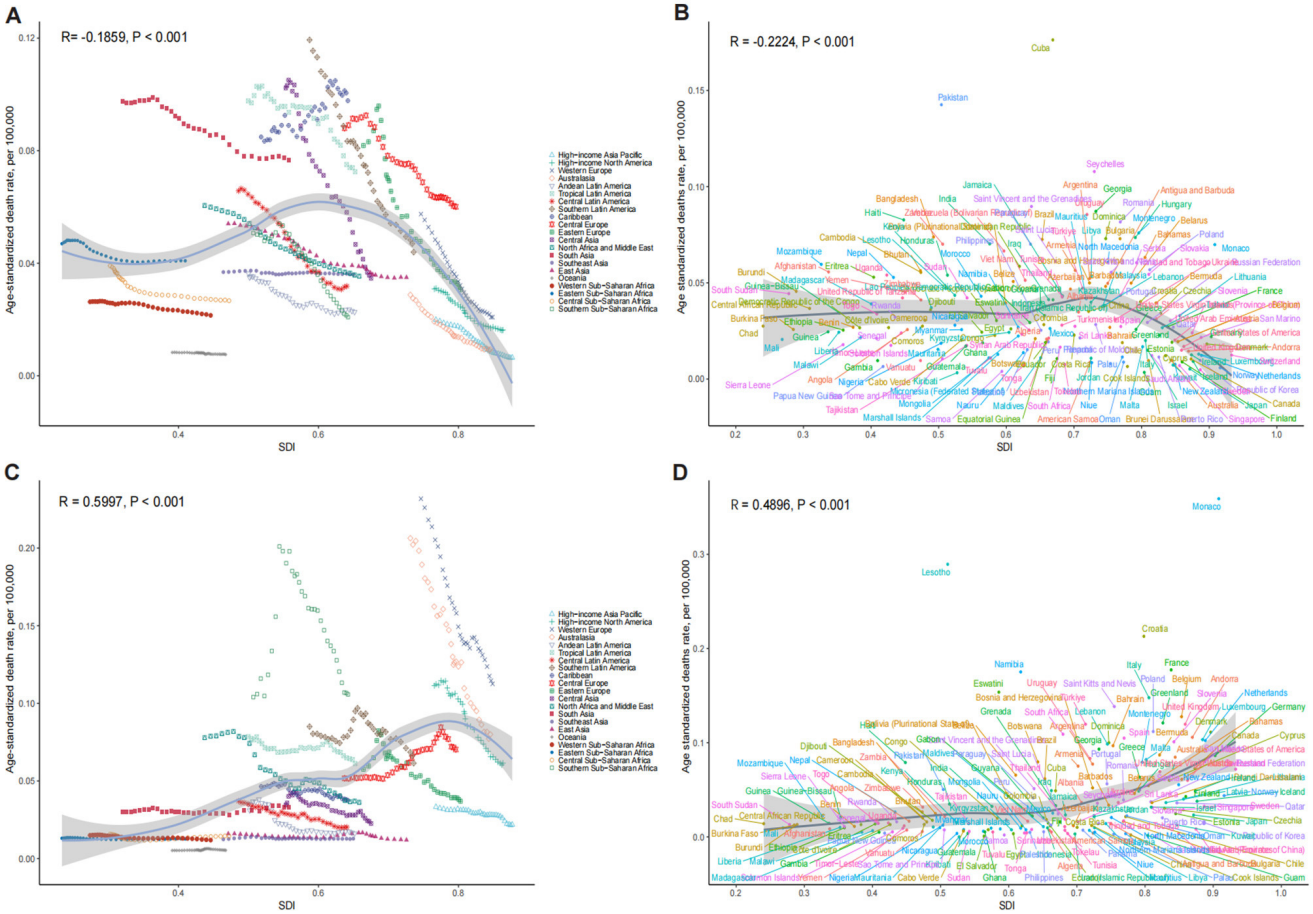
The association between SDI and ASDR of LC attributable to OESA **(A, B)** and OEA **(C, D)**. **(A, C)** Trends in 21 regions, 1990-2021; **(B, D)** Estimates for 204 countries, 2021. The blue line represents the expected ASDR of LC based solely on SDI. SDI, socio-demographic index; ASDR, age-standardized death rate; LC, laryngeal cancer; OESA, occupational exposure to sulfuric acid; OEA, occupational exposure to asbestos.

The ASDR for OESA declined in all SDI regions, with the greatest reduction in high-middle SDI regions from 0.07 (95% UI: 0.03–0.14) in 1990 to 0.03 (95% UI: 0.01–0.07) in 2021 (Table 1 and Figure 3A). The ASDR for OEA decreased in the high, high-middle, and middle SDI regions, with the greatest decrease in the high-middle SDI regions from 0.10 (95% UI: 0.05–0.14) in 1990 to 0.04 (95% UI: 0.02–0.07) in 2021 (Table 2 and Figure 3B). The ASDR for OESA showed a negative correlation with SDI (*R* = −0.1859, *p* < 0.001), peaking at an SDI of ~0.6 before declining SDI became >0.6 (Figure 4A). Conversely, the ASDR for OEA showed a positive correlation with SDI (*R* = 0.5997, *p* < 0.001), before declining when SDI became >0.8. The ASDR increased at SDI < 0.55, and 0.65 < SDI < 0.8 (Figure 4C).

**Figure 5:**
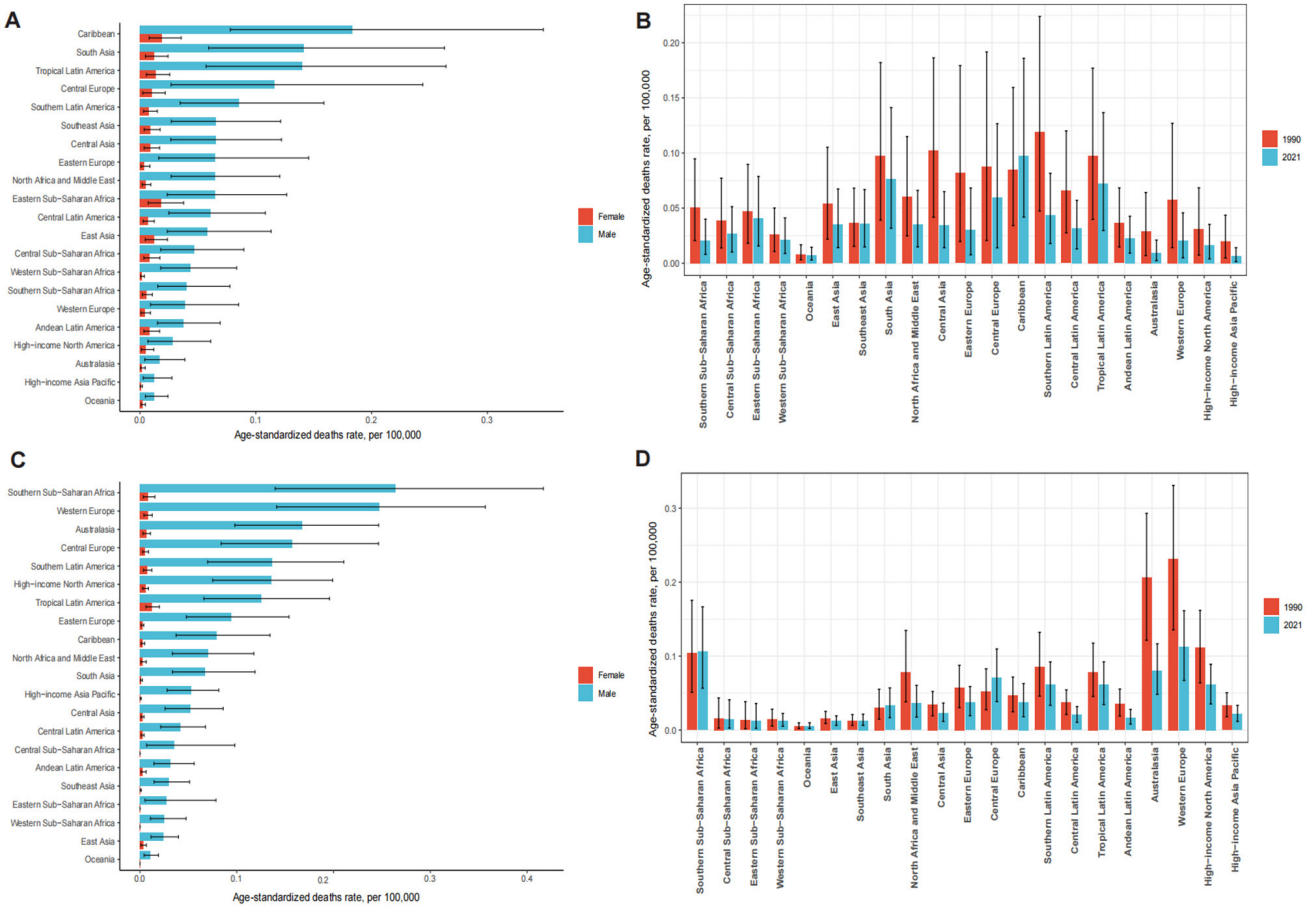
The ASDR of LC attributable to OESA **(A)** and OEA **(C)** by sex among 21 regions in 2021. The ASDR of LC attributable to OESA **(B)** and OEA **(D)** among 21 regions in 1990 and 2021. ASDR, age-standardized death rate; LC, laryngeal cancer; OESA, occupational exposure to sulfuric acid; OEA, occupational exposure to asbestos.

In 1990, the highest OESA-related deaths in South Asia, East Asia, Western Europe, Eastern Europe, and Central Europe, with South Asia having the largest burden (663.77, 95% UI: 265.92–1,235.79). In 2021, South Asia remained the most affected (1,243.55, 95% UI: 516.03–2,290.45), followed by East Asia, Southeast Asia, Tropical Latin America, North Africa and the Middle East. Among all regions, ASDR only increased in the Caribbean (from 0.08 [95% UI: 0.03–0.16] in 1990 to 0.10 [95% UI: 0.04–0.19] in 2021); EAPC: 0.71[95% UI: 0.59–0.83]), while Eastern Europe showed the greatest decline (from 0.08 [95% UI: 0.02–0.18] in 1990 to 0.03 [95% UI: 0.01–0.07] in 2021; Figures 5A, B and Table 1).

Western Europe had the highest number of OEA-related deaths in 1990 (1,407.76, 95% UI: 826.25–2,012.62) and 2021 (1,178.05, 95% UI: 700.44–1,673.87). South Asia showed an evident increase in the number of deaths from 149.34 (95% UI: 71.42–275.60) in 1990 to 442.21(95% UI: 222.86–764.82) in 2021. The highest ASDR in 2021 was observed in males across 21 regions, with declining trends, except in Central Europe, where rates remained stable. Australasia had the largest decline in ASDR from 0.21 (95% UI: 0.12–0.29) in 1990 to 0.08 (95% UI: 0.05–0.12) in 2021 (Figures 5C, D and Table 2).

### 3.3 Trends by national-level SDI

**Figure 6:**
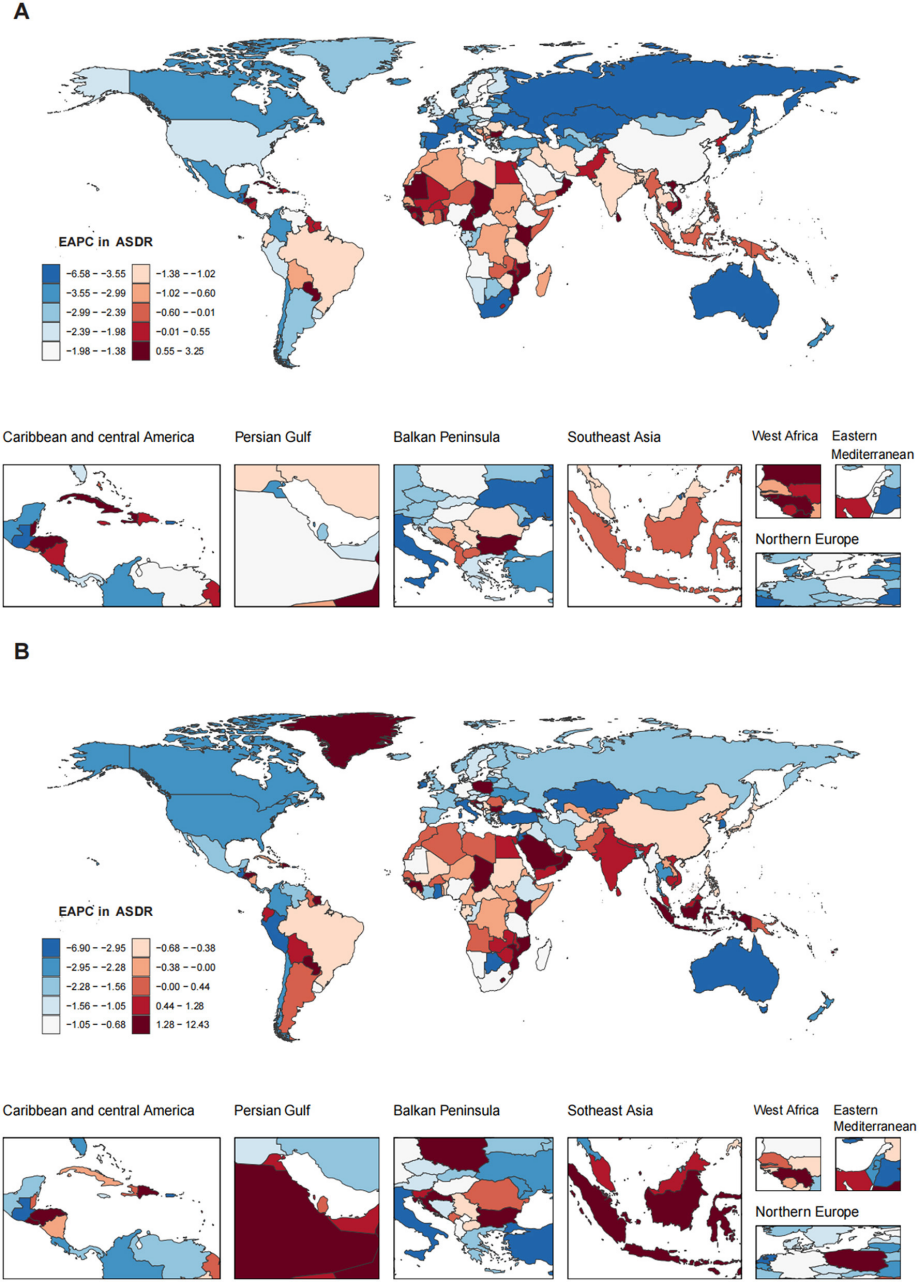
The EAPC in ASDR of LC attributable to OESA **(A)** and OEA **(B)** among 204 countries and territories from 1990 to 2021. Heat gradient represents deciles from red (highest) to blue (lowest). EAPC, estimated annual percentage change; ASDR, age-standardized death rate; LC, laryngeal cancer; OESA, occupational exposure to sulfuric acid; OEA, occupational exposure to asbestos.

Nationally, the ASDR for OESA showed a negative correlation with SDI (*R* = −0.2224, *p* < 0.001), peaking at an SDI of ~0.72 before declining when SDI became > 0.72. Cuba had the highest ASDR (0.18, 95% UI: 0.07–0.34), followed by Pakistan, Seychelles, Saint Vincent and the Grenadines, and Georgia (Figure 4B and Supplementary Table S1). The greatest increases in ASDR were observed in Kiribati, the Solomon Islands, Sri Lanka, Chad, and Guinea, while the Republic of Korea, Singapore, Estonia, Spain, and Kazakhstan exhibited the largest declines (Figure 6A and Supplementary Table S1).

The ASDR for OEA showed a positive correlation with SDI (*R* = 0.4900, *p* < 0.001) and increased with SDI. Monaco had the highest ASDR (0.36, 95% UI: 0.19–0.60), followed by Lesotho, Croatia, France, and Namibia (Figure 4D and Supplementary Table S1). The greatest increases in ASDR were observed in Georgia, Greenland, Saudi Arabia, Oman, and El Salvador, while Guam, American Samoa, Singapore, Peru, and Kazakhstan exhibited the largest declines (Figure 6B and Supplementary Table S1).

### 3.4 Decomposition analysis

**Figure 7:**
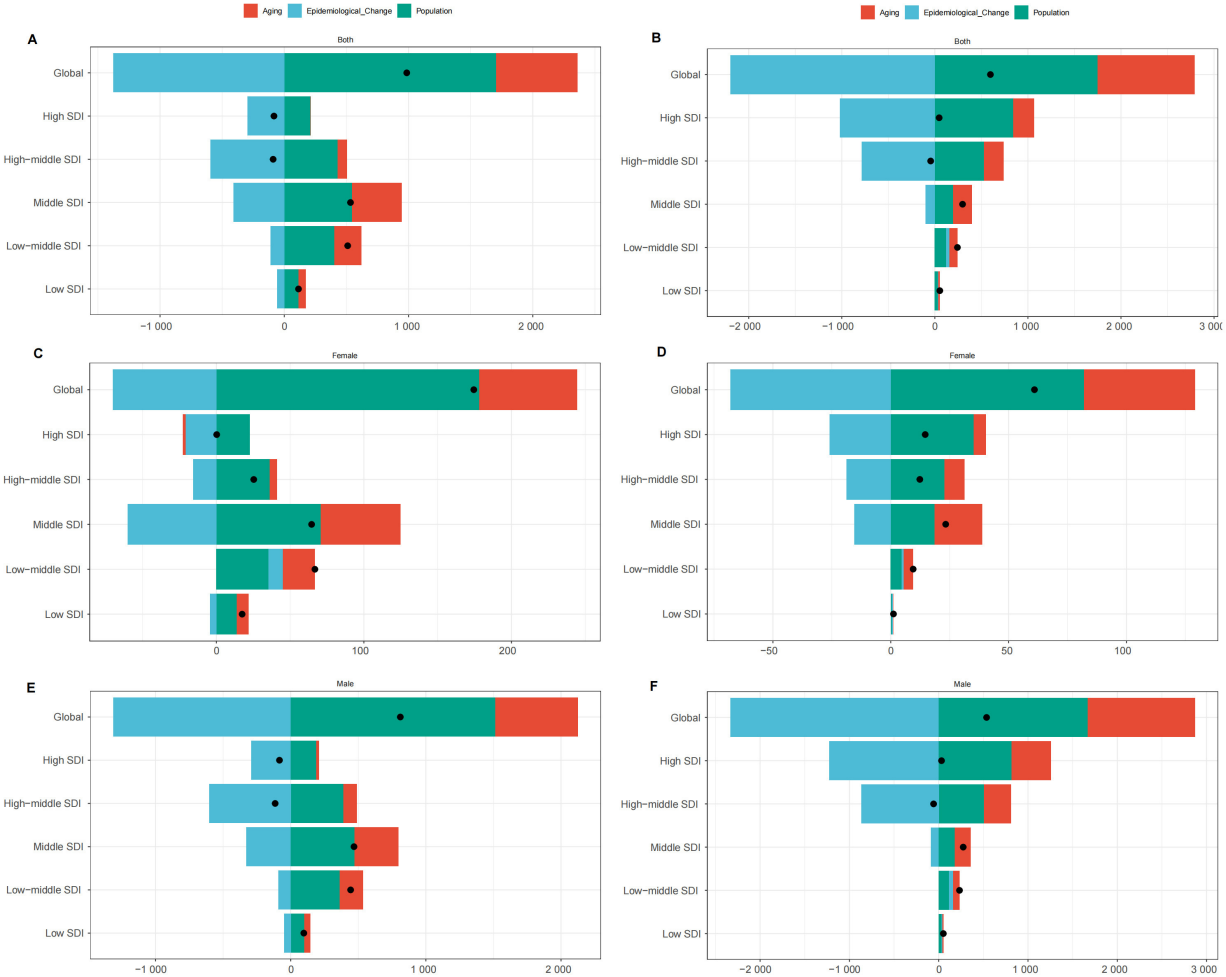
Changes in LC deaths attributable to OESA [**(A)** overall, **(C)** females, **(E)** males] and OEA [**(B)** overall, **(D)** females, **(F)** males], 1990–2021. Analyses by global/SDI quintile and drivers (population growth, aging, epidemiology). Black dots represent the total change contributed by all three components. A positive value for each component indicates a positive contribution to LC deaths, and a negative value indicates a negative contribution. LC, laryngeal cancer; OESA, occupational exposure to sulfuric acid; OEA, occupational exposure to asbestos; SDI, socio-demographic index.

To assess the impact of population growth, aging, and epidemiological changes on LC deaths attributable to OESA and OEA from 1990 to 2021, we conducted a decomposition analysis (Figure 7). Globally, LC deaths attributable to OESA have increased over the past 32 years, particularly in the middle, low-middle, and low SDI regions, while the high and high-middle SDI regions showed a slight decline. The increase was primarily driven by population growth and aging, accounting for 172.75 and 66.81% of the change, respectively. In contrast, decline was driven by epidemiological changes, particularly in the high-middle (−355.42%) and high SDI regions (−661.72%). As female deaths were markedly lower than male deaths, global and SDI-specific trends closely followed those observed in males (Figures 7A, C, E and Supplementary Table S2).

Similarly, OEA-related LC deaths also increased globally, largely due to population growth (292.53%) and aging (175.2%). In the low-middle and low SDI regions, all three factors—population growth, aging, and epidemiological changes—contributed to an increase in deaths. In high and high-middle SDI regions, aging (486.77 and 481.93%, respectively) and population growth (1812.37 and 1168.66%, respectively) contributed to rising deaths, but epidemiological improvements led to an overall decline (−2199.14 and −1750.59%, respectively). Global trends for OEA also followed those in males (Figures 7B, D, F and Supplementary Table S3).

### 3.5 Frontier analysis

**Figure 8:**
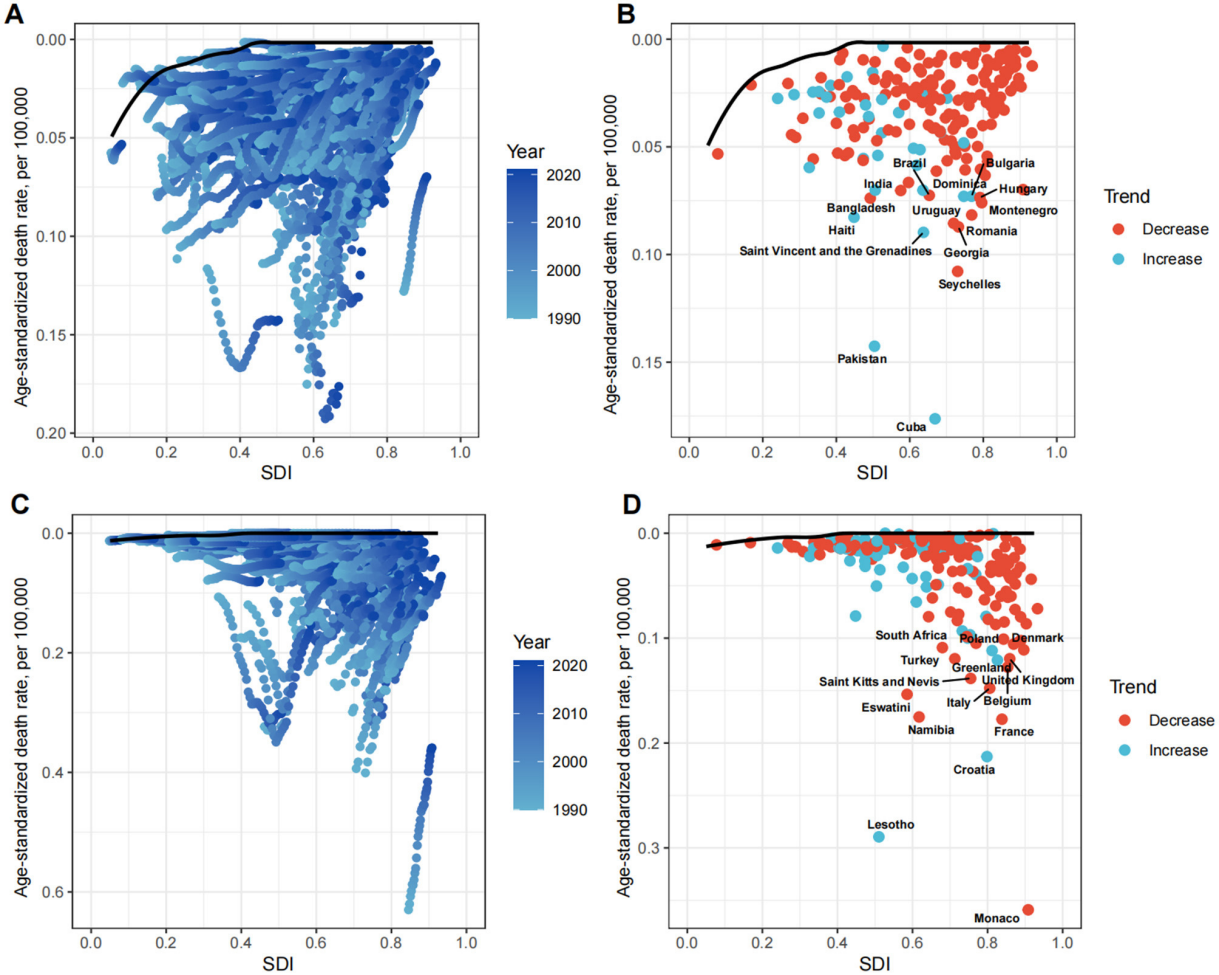
Frontier analysis exploring the relationship between SDI and ASDR for LC attributable to OESA **(A, B)** and OEA **(C, D)** in 204 countries and territories. In figures **(A, C)** light blue (1990) to dark blue (2021) indicates the change in years. The frontier line delineates the countries and territories with lowest ASDR (optimal performers) given their SDI. In figures **(B, D)** each point represents a specific country or territory in 2021, the frontier line is shown in black, and the top 15 countries and territories with the largest differences from the frontier are marked in black. The direction of ASDR change from 1990 to 2021 is indicated by the color of the dots, with red dots representing decreases and blue dots representing increases. SDI, socio-demographic index; ASDR, age-standardized death rate; LC, laryngeal cancer; OESA, occupational exposure to sulfuric acid; OEA, occupational exposure to asbestos.

To explore possible reductions in the ASDR, frontier analysis was performed using SDI as a factor. The effective difference for a given SDI generally decreased with an increasing global SDI (Figure 8).

For OESA, the 15 countries with the largest actual differences (effective difference range: 0.07–0.18 per 100,000 population) included Cuba, Pakistan, Seychelles, Georgia, Romania, Montenegro, Hungary, Bulgaria, Dominica, Uruguay, Brazil, India, Bangladesh, Haiti, and Saint Vincent and the Grenadines. Notably, these countries are not located in high SDI regions. Countries in the high-middle SDI regions (Seychelles, Georgia, Romania, Montenegro, Hungary, Bulgaria, Dominica, and Uruguay) showed the highest potential for reducing the LC death burden attributable to OESA (Figure 8B and Supplementary Table S4). For OEA, the 15 countries with the largest actual differences (effective difference range: 0.11–0.36 per 100,000 population) included Monaco, Lesotho, Namibia, Eswatini, Saint Kitts and Nevis, Turkey, South Africa, Croatia, France, Belgium, Italy, the United Kingdom, Greenland, Poland, and Denmark. Countries in high SDI regions (Monaco, France, Belgium, Italy, the United Kingdom, Greenland, Poland, and Denmark) demonstrated showed the highest potential for reducing the LC death burden attributable to OEA (Figure 8D and Supplementary Table S4).

### 3.6 Predicted trends of LC attributable to OESA and OEA till 2040

**Figure 9:**
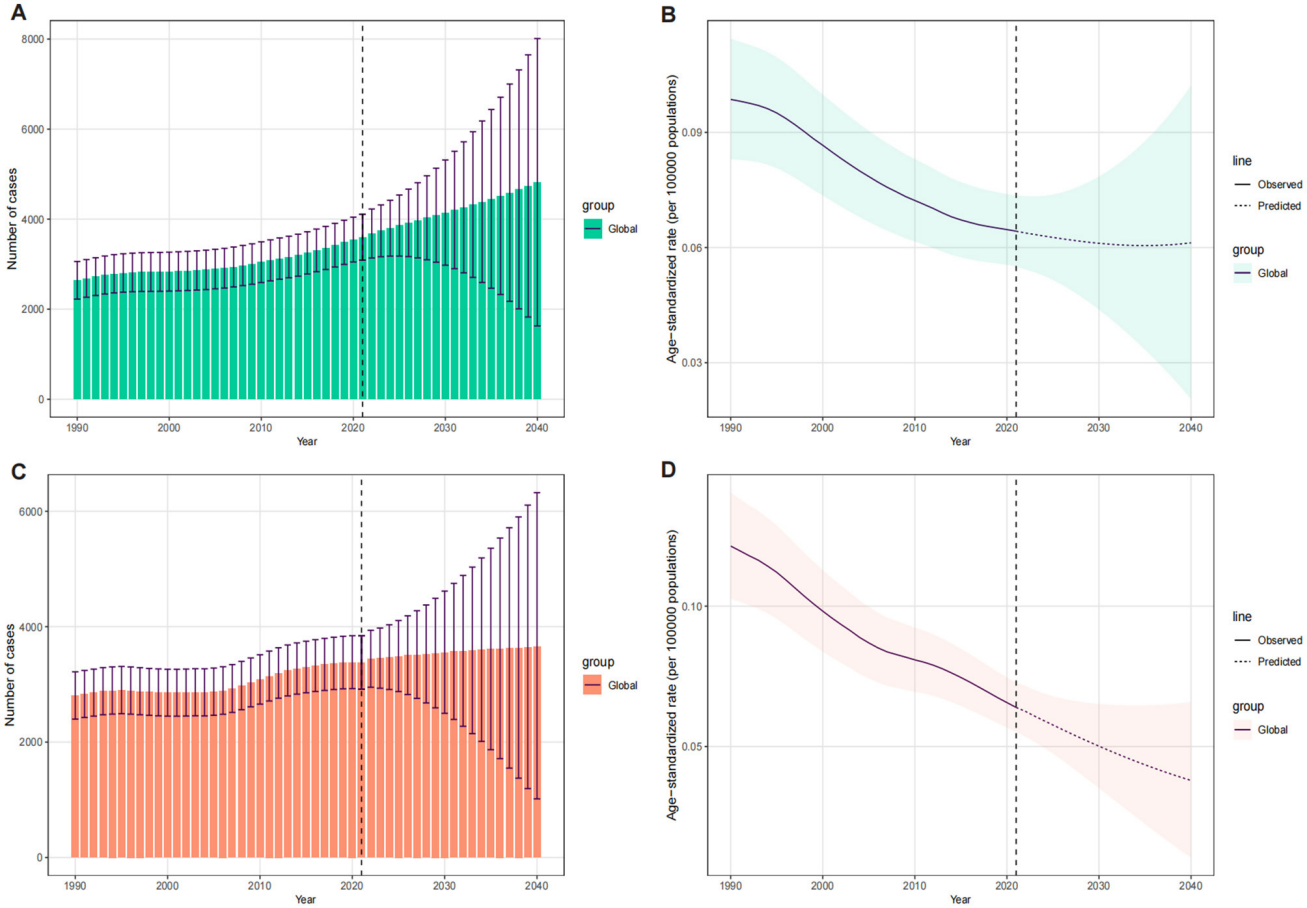
The temporal trends of number of deaths and ASDR for LC attributable to OESA [**(A)** deaths, **(B)** ASDR] and OEA [**(C)** deaths, **(D)** ASDR], 1990–2040, globally. The vertical dashed line indicates where prediction starts. The fan shows the predictive distribution between the 5 and 95% quantile, whereby the shaded bands show prediction intervals in increments of 10%. ASDR, age-standardized death rate; LC, laryngeal cancer; OESA, occupational exposures to sulfuric acid; OEA, occupational exposures to asbestos.

The BAPC model was used to predict future trends in LC deaths and ASDR attributable to OESA and OEA up to 2040 (Figure 9). The results indicated a gradual increase in the overall LC deaths attributable to OESA and OEA from 2022 to 2040. Deaths attributable to OESA are projected to increase from 3,679.08 (95% UI: 3,137.17–4,225.18) in 2022 to 4,810.90 (95% UI: 1,628.04–8,010.74) in 2040, while deaths attributable to OEA are projected to increase from 3,441.53 (95% UI: 2,950.53–3,937.46) in 2022 to 3,648.48 (95% UI: 1,016.75–6,322.42) in 2040 (Figures 9A, C and Supplementary Table S5). The ASDR for OESA is projected to remain stable at 0.06 (95% UI: 0.05–0.07 in 2022; 95% UI: 0.02–0.10 in 2040), while the ASDR for OEA is projected to decline from 0.06 (95% UI: 0.05–0.07) in 2022 to 0.04 (95% UI: 0.01–0.07) in 2040 (Figures 9B, D and Supplementary Table S5).

## 4 Discussion

To the best of our knowledge, this is the first study to investigate the death burden and trends of LC attributable to OESA and OEA at the global, regional, and national levels. This study provides a comprehensive analysis of epidemiological trends in LC death burdens attributable to OESA and OEA from 1990 to 2021, considering factors such as sex, age, and SDI. We observed an overall increase in LC deaths attributable to OESA and OEA, despite a downward trend in ASDR from 1990 to 2021. This trend is consistent with the global decline in LC mortality rates over the same period (1, 2).

A significant negative correlation was observed between ASDR for OESA and SDI at the regional level from 1990 to 2021. Deaths decreased in the high-middle and high SDI regions, and increased in the middle, low-middle, and low SDI regions. In the high and high-middle SDI regions, many chemical factories associated with sulfuric acid mists operated before 1990. Following the identification of sulfuric acid mist as a carcinogen, developed countries may have taken measures that led to a decrease in death rates by implementing regulations and policies in the 1970s−1990s, such as setting occupational exposure limits and dismantling sulfuric acid production facilities (25). Furthermore, the development of larynx-preserving strategies and the widespread use of endoscopy have indirectly reduced LC mortality. A higher economic status also facilitate earlier diagnosis and effective treatment (2, 26–28). Global growth in industrialization in middle, low-middle, and low SDI regions (29–31), coupled with the outsourcing of occupational hazards from developed countries to developing countries (32), may have contributed to rising death rates. Less-developed regions often face challenges such as inadequate healthcare coverage, scarce medical resources, lack of public health awareness, and lack of early cancer screening implementation (2, 33).

Our study revealed a significant positive correlation between ASDR for OEA and SDI at the regional level from 1990 to 2021. The number of deaths and ASDR were highest in the high SDI regions, likely due to asbestos exposure in the 1960s and 1970s during the peak industrial period (34). The ASDR decreased in high, high-middle, and middle SDI regions but increased in low-middle and low SDI regions. Many developed countries have implemented strict restrictions on the production, importation, and use of asbestos. Evidence from several developed countries shows that asbestos-related deaths have declined in cohorts born after the 1940s (35–37). In contrast, with the continued industrialization and widespread use of asbestos in low-middle and low SDI regions (14, 38), the ASDR for OEA has increased. The ongoing risks of OEA remain poorly understood in several developing countries and mitigation strategies often lack public attention (39).

Regionally, South Asia, East Asia and Southeast Asia accounted for the highest OESA-related LC deaths in 2021, while Western Europe, Eastern Europe, and Central Europe experienced declines from 1990 to 2021. These results highlight the need to reduce OESA-related LC burden, particularly in Asia. The Caribbean had the highest ASDR in 2021 and experienced the highest increase in ASDR from 1990 to 2021, with an EAPC of 0.71. Although strategies have been developed for occupational health problems in the Caribbean, such as Latin America and the Caribbean Code Against Cancer (15), the ASDR for OESA continues to rise. Strengthening occupational exposure limits, dismantling sulfuric acid production facilities, and enhancing investments in primary healthcare resources may help reduce the death burden regionally. Europe remains a major contributor to asbestos-related diseases burden (40). The number of OEA-related LC deaths in Europe was higher than in other continents, particularly in Western Europe. Although the ASDR in Western Europe has decreased, regulations on asbestos production and use are still necessary. Australasia experienced the largest decline in ASDR, largely due to manufacturing unions advocating for policy changes, including the complete ban of asbestos in 2003 (41).

Nationally, the ASDR for OESA showed a notable negative correlation with SDI, while a positive correlation was observed for OEA. For OESA, Cuba, Pakistan, Seychelles, and Georgia had the highest ASDR of LC attributable to OESA, whereas Kiribati, Solomon Islands, Sri Lanka, and Chad had the highest increases in ASDR. Based on the frontier analysis, countries such as Seychelles, Georgia, and Romania, which have relatively high SDI levels, should prioritize the implementation of more effective mitigation measures. For OEA, Monaco, Lesotho, Croatia, and France had the highest ASDR, whereas Georgia, Greenland, Saudi Arabia, and Oman had the highest significant increases in ASDR. High SDI countries, such as Monaco, France, Italy, and Belgium, should review and strengthen their occupational health policies.

In 2021, the global death burden of LC attributable to OESA and OEA were higher in males compared to females. Males are more likely to work in higher-exposure occupations, such as mining, construction, and certain chemical manufacturing. Although the number of deaths among females is lower, OESA and OEA in women remains a significant public health concern that warrants attention (42). In both sexes, LC deaths attributable to OESA and OEA increased with age, possibly due to prolonged occupational exposure in youth. Additionally, lower immunity, further complications, and higher nutritional needs in the elderly may also contribute to increased mortality rates (43).

Decomposition analysis revealed that the impact of population growth, aging, and epidemiological changes on the death burden varied across regions with different SDI. Population growth and aging were the primary contributors to increasing deaths in middle, low-middle, and low SDI regions. In contrast, in high-middle and high SDI regions, epidemiological changes contributed to reducing the death burden. These findings suggest that tailored strategies are required to address the disease burden in different regions. In less-developed regions, efforts should focus on mitigating the challenges posed by population growth and aging, such as improving healthcare infrastructure and early detection programs. In more developed regions, efforts to sustain and enhance the positive effects of epidemiological changes are needed, and stricter regulations on occupational exposure may be required.

The BAPC model indicated that the overall number of LC deaths attributable to OESA and OEA will gradually increase from 2022 to 2040. The health risks posed by sulfuric acid and asbestos remain significant, but can be improved through health policies. In high and high-middle SDI regions, the LC deaths and ASDR have been effectively controlled due to the promulgation of regulations reducing OESA and OEA, as well as the implementation of governance measures. In contrast, low and low-middle SDI regions and countries face a growing LC burden, and less-developed regions should learn relevant policies and experiences from developed regions. Furthermore, developing alternative materials to replace asbestos and improving production processes to reduce sulfuric acid exposure are crucial. Strengthening international cooperation in sharing best practices between countries is essential for reducing the global disease burden.

Our study has some limitations. First, although the GBD 2021 study made extensive efforts to collect all available data, limited data from some countries may have led to an underestimation of the burden and trends of LC attributable to OESA and OEA. Second, racial factors that could significantly influence disease burden, were not accounted for in the GBD datasets. Third, the diagnosis of LC is likely due to the absence of symptoms that reduce the likelihood of seeking medical attention, such as hoarseness, dyspnea, and dysphagia, which may affect the accuracy of mortality statistics. Further studies addressing these limitations are necessary to validate our findings.

## 5 Conclusion

In conclusion, although the ASDR decreased from 1990 to 2021, OESA and OEA remain contributors to LC mortality worldwide, with the number of deaths projected to increase over the next two decades. Disease burden varied significantly across SDI regions, suggesting that preventive measures should be tailored to specific SDI levels. Public health policies should be proactive, focusing on enforcing regulations on OESA and OEA. More effective health interventions and efficient preventative strategies should be established to reduce the LC burden attributable to OESA and OEA.

## Data Availability

The original contributions presented in the study are included in the article/Supplementary material, further inquiries can be directed to the corresponding author.

## References

[1] Huang J Chan SC Ko S Lok V Zhang L Lin X. Updated disease distributions, risk factors, and trends of laryngeal cancer: a global analysis of cancer registries. Int J Surg. (2024) 110:810–9. 10.1097/JS9.0000000000000902 38000050 PMC10871644

[2] Zhou T Wang X Zhu Q Zhou E Zhang J Song F. Global trends and risk factors of laryngeal cancer: a systematic analysis for the Global Burden of Disease Study (1990-2021). BMC Cancer. (2025) 25:296. 10.1186/s12885-025-13700-4 39972455 PMC11837445

[3] Koroulakis A Agarwal M. Laryngeal Cancer. In: StatPearls [Internet]. (Treasure Island, FL: StatPearls Publishing) (2025). 30252332

[4] Steuer CE El-Deiry M Parks JR Higgins KA Saba NF. An update on larynx cancer. CA Cancer J Clin. (2017) 67:31–50. 10.3322/caac.21386 27898173

[5] Jha S Sinha S Mahadevappa P Hazra S Sarka S. Assessing water quality and human health risk near coal mines and industrial area of Singrauli, India: special emphasis on toxic elements. Environ Geochem Health. (2024) 46:449. s10653-024-02235-5 10.1007/s10653-024-02235-5 39316161

[6] Sandhu APS. Tanvir Singh K Singh S Antaal H Luthra S. Decoding cancer risk: understanding gene-environment interactions in cancer development. Cureus. (2024) 16:e64936. 10.7759/cureus.64936 39165474 PMC11335134

[7] Miao X Yao T Dong C Chen Z Wei W Shi Z. Global, regional, and national burden of non-communicable diseases attributable to occupational asbestos exposure 1990-2019 and prediction to 2035: worsening or improving? BMC Public Health. (2024) 24:832. 10.1186/s12889-024-18099-4 38500093 PMC10946175

[8] IARC monographs on the evaluation of carcinogenic risks to humans. Chemical agents and related occupations. Lion: International Agency for Research on Cancer. (2012) 100:336. PMC4781612 23189753

[9] Yang JH Koedrith P. Kang DS Kee NK Jung JH Lee CM. A putative adverse outcome pathway relevant to carcinogenicity induced by sulfuric acid in strong inorganic acid mists. J Cancer Prev. (2019) 24:139–45. 10.15430/JCP.2019.24.3.139 31624719 PMC6786810

[10] Steenland K. Laryngeal cancer incidence among workers exposed to acid mists (United States). Cancer Cause Control. (1997) 8:34–8. 10.1023/A:1018427003878 9051320

[11] International Agency for Research on Cancer (IARC). Asbestos. In Arsenic, Metals, Fibres and Dusts. IARC Monographs on the Evaluation of Carcinogenic Risks to Humans (Lyon, France: IARC) (2012).Volume 100C, pp. 219-309. PMC4781271 23189751

[12] Clin B Gramond C Thaon I Brochard P Delva F Chammings S. Head and neck cancer and asbestos exposure. Occup Environ Med. (2022) 79:690–6. 10.1136/oemed-2021-108047 35393288 PMC9484389

[13] Peng WJ Mi J Jiang YH. Asbestos exposure and laryngeal cancer mortality. Laryngoscope. (2015) 126:1169–74. 10.1002/lary.25693 26418833

[14] Lin C Cheng W Liu X Li H Song Y. The global, regional, national burden of laryngeal cancer and its attributable risk factors (1990-2019) and predictions to 2035. Eur J Cancer Care. (2022) 31:e13689. 10.1111/ecc.13689 35980023

[15] Estela B Eduardo A Luis AC López-Carrillo L Mora AM Rodríguez-Guzmán J. Latin America and the Caribbean Code Against cancer 1st edition: environment, occupation, and cancer. Cancer Epidemiol. (2023) 1:102381. 10.1016/j.canep.2023.102381 37852723 PMC13094079

[16] Chen T Sun XM Wu L. High time for Complete Ban on asbestos use in developing countries. JAMA Oncol. (2019) 5:779–80. 10.1001/jamaoncol.2019.0446 31120531

[17] Takala J. Eliminating occupational cancer. Ind Health. (2015) 53:307–9. 10.2486/indhealth.53-307 26377441 PMC4551060

[18] GBD 2021 Risk Factors Collaborators. Global burden and strength of evidence for 88 risk factors in 204 countries and 811 subnational locations, 1990-2021: a systematic analysis for the Global Burden of Disease Study 2021. Lancet. (2024) 403:2162–203. 10.1016/S0140-6736(24)00933-4 38762324 PMC11120204

[19] Global Burden of Disease Collaborative Network. Global Burden of Disease Study 2021 (GBD 2021) Socio-Demographic Index (SDI) 1950–2021. Seattle, United States of America: Institute for Health Metrics and Evaluation (IHME), 2024.

[20] Deng Y Zhao P Zhou L Xiang D Hu J Liu Y. Epidemiological trends of tracheal, bronchus, and lung cancer at the global, regional, and national levels: a population-based study. J Hematol Oncol. (2020) 13:98. 10.1186/s13045-020-00915-0 32690044 PMC7370495

[21] Chevan A Sutherland M. Revisiting Das Gupta: refinement and extension of standardization and decomposition. Demography. (2009) 46:429–49. 10.1353/dem.0.0060 19771938 PMC2831344

[22] Xie Y Bowe B Mokdad AH Xian H Yan Y Li T. Analysis of the global burden of disease study highlights the global, regional, and national trends of chronic kidney disease epidemiology from 1990 to 2016. Kidney Int. (2018) 94:567–81. 10.1016/j.kint.2018.04.011 30078514

[23] Knoll M Furkel J Debus J Abdollahi A Karch A Stock C. An R package for an integrated evaluation of statistical approaches to cancer incidence projection. BMC Med Res Methodol. (2020) 20:257. 10.1186/s12874-020-01133-5 33059585 PMC7559591

[24] Stevens GA Alkema L Black RE Boerma JT Collins GS Ezzati M. Guidelines for accurate and transparent health estimates reporting: the GATHER statement. Lancet. (2016) 388:e19–23. 10.1016/S0140-6736(16)30388-9 27371184

[25] Occupational exposures to mists and vapours from strong inorganic acids and other industrial chemicals. Working Group views and expert opinions, Lyon, 15-22 October 1991. IARC Monogr Eval Carcinog Risks Hum. (1992) 54, 1–310. 1345371 PMC5366853

[26] Forastiere AA Ismaila N Lewin JS Nathan CA Adelstein DJ Eisbruch A. Use of Larynx-preservation strategies in the treatment of laryngeal cancer: American society of clinical oncology clinical practice guideline update. J Clin Oncol. (2018) 36:1143–69. 10.1200/JCO.2017.75.7385 29172863

[27] Qasem M Qasem N Kinshuck A Milinis K. Long-term laryngeal function and quality of life following treatment of early glottic cancer: a meta-analysis. Otolaryngol Head Neck Surg. (2025) 172:375–85. 10.1002/ohn.1011 39403814

[28] Barbalata C Mattos LS. Laryngeal tumor detection and classification in endoscopic video. IEEE J Biomed Health Inform. (2016) 20:322–32. 10.1109/JBHI.2014.2374975 25438330

[29] Pearce N Matos E Boffetta P Kogevinas M Vainio H. Occupational exposures to carcinogens in developing countries. Ann Acad Med Singap. (1994) 23:684–9. 7847748

[30] Kumie A Amera T Berhane K Samet J Hundal N. G/Michael F. Occupational health and safety in Ethiopia: a review of situational analysis and needs assessment. Ethiop J Health Dev. (2016) 30:17–27. 28867918 PMC5578617

[31] Yang M A. current global view of environmental and occupational cancers. J Environ Sci Health C. (2011) 29:223–49. 10.1080/10590501.2011.601848 21929381

[32] Park J Hisanaga N Kim Y. Transfer of occupational health problems from a developed to a developing country: lessons from the Japan-South Korea experience. Am J Ind Med. (2009) 52:625–32. 10.1002/ajim.20723 19562727

[33] Lebo NL Khalil D Balram A Holland M Corsten M Ted McDonald J. Influence of Socioeconomic Status on Stage at Presentation of Laryngeal Cancer in the United States. Otolaryngol Head Neck Surg. (2019) 161:800–6. 10.1177/0194599819856305 31184265

[34] Virta RL. Worldwide asbestos supply and consumption trends from 1900 through 2003. U.S. Geological Survey Circular (2006) 1298. 10.3133/cir1298

[35] Boffetta P Malvezzi M Pira E Negri E La Vecchia C. International analysis of age-specific mortality rates from mesothelioma on the basis of the international classification of diseases, 10th revision. J. Global Oncol. (2017) 4:1–15. 10.1200/JGO.2017.010116 30241199 PMC6180783

[36] Segura O Burdorf A Looman C. Update of predictions of mortality from pleural mesothelioma in the Netherlands. Occup Environ Med. (2003) 60:50–6. 10.1136/oem.60.1.50 12499457 PMC1740374

[37] Magnani C Mensi C Binazzi A Marsili D Grosso F Ramos-Bonilla JP. The Italian experience in the development of mesothelioma registries: a pathway for other countries to address the negative legacy of Asbestos. Int J Environ Res Public Health. (2023) 20:936. 10.3390/ijerph20020936 36673690 PMC9858856

[38] Ramos-Bonilla JP Giraldo M Marsili D Pasetto R Terracini B Mazzeo A. An approach to overcome the limitations of surveillance of asbestos related diseases in low- and middle-income countries: what we learned from the Sibaté Study in Colombia. Ann Glob Health. (2023) 89:64. 10.5334/aogh.4166 37810608 PMC10558025

[39] Douglas T Van den Borre L. Asbestos neglect: why asbestos exposure deserves greater policy attention. Health Policy. (2019) 123:516–9. 10.1016/j.healthpol.2019.02.001 30770142

[40] Kameda T Takahashi K Kim R Jiang Y Movahed M Park EK. Asbestos: use, bans and disease burden in Europe. Bull World Health Organ. (2014) 92:790–7. 10.2471/BLT.13.132118 25378740 PMC4221761

[41] Soeberg M Vallance DA Keena V Takahashi K Leigh J. Australia's Ongoing legacy of asbestos: significant challenges remain even after the complete banning of asbestos almost fifteen years ago. Int J Env Res Pub Health. (2018) 15:384. 10.3390/ijerph15020384 29473898 PMC5858453

[42] Hall AL Kromhout H Schüz J Peters S Portengen L Vermeulen R. Laryngeal cancer risks in workers exposed to lung carcinogens: exposure-effect analyses using a quantitative job exposure matrix. Epidemiology. (2020) 31:145–54. 10.1097/EDE.0000000000001120 31577634

[43] Montégut L López-Otín C Kroemer G. Aging and cancer. Mol Cancer. (2024) 23:106. 10.1186/s12943-024-02020-z 38760832 PMC11102267

