# Supplementary material for: Global, regional and national mortality burden of laryngeal cancer attributable to occupational exposure to sulfuric acid and asbestos: 1990–2021 and projections to 2040
Source: Front Public Health. 2025 Jul 18;13:1602789. doi: 10.3389/fpubh.2025.1602789 (PMC12315700; doi:10.3389/fpubh.2025.1602789)
Supplement: Supplementary file 1 [file Data_Sheet_1.pdf]

Supplementary Material

Supplementary Table 1 Top five countries and territories with the Highest ASDR in 2021, the Highest and lowest EAPC from 1990 to 2021 of laryngeal cancer atrributable to OESA and OEA at the national level.

	**OESA**			**OEA**		
	**ASDR in 2021 per 100,000, 95%UI**	**the Highest EAPC, %,**	**The lowest EAPC, %,**	**ASDR in 2021 per 100,000, 95%UI**	**the Highest EAPC, %,**	**The lowest EAPC, %,**
Countries and territories						
1st	Cuba (0.18, [0.07, 0.34])	Kiribati (3.22)	Republic of Korea (-6.51)	Monaco (0.36, [0.19, 0.60])	Georgia (12.31)	Guam (-6.83)
2nd	Pakistan (0.14, [0.06, 0.27])	Solomon Islands (3.06)	Singapore (-5.06)	Lesotho (0.29, [0.11, 0.52])	Greenland (8.66)	American Samoa (-6.58)
3rd	Seychelles (0.11, [0.04, 0.20])	Sri Lanka (2.65)	Estonia (-4.92)	Croatia (0.21, [0.11, 0.34])	Saudi Arabia (7.15)	Singapore (-5.77)
4th	Saint Vincent and the Grenadines (0.09, [0.04, 0.16])	Chad (2.14)	Spain (-4.65)	France (0.18, [0.10, 0.26])	Oman (4.35)	Peru (-5.27)
5th	Georgia (0.09, [0.04 0.17])	Guinea (2.09)	Kazakhstan (-4.63)	Namibia (0.18, [0.08, 0.31])	El Salvador (4.2)	Kazakhstan (-4.93)

Abbreviations: ASDR = age standardized death rate; OESA=occupational exposure to sulfuric acid asbestos ; OEA=occupational exposure to asbestos; EAPC = estimated annual percentage change; UI: uncertainty intervals.

Supplementary Table 2 Changes in deaths of laryngeal cancer atrributable to OESA according to decomposition analysis from 1990 to 2021 in global and SDI regional level.

	**Overall**			**Population growth**			**Aging**			**Epidemiological changes**		
	**both**	**male**	**female**	**both**	**male**	**female**	**both**	**male**	**female**	**both**	**male**	**female**
Global	985.65	811.14	174.51	1702.68(172.75%)	1513.12(186.54%)	178.26(102.15%)	658.53(66.81%)	610.23(75.23%)	66.38(38.04%)	-1375.55(-139.56%)	-1312.21(-161.77%)	-70.13(-40.19%)
SDI regions												
high	-82.99	-83.2	0.21	210.73(253.91%)	187.61(225.48%)	22.77(10822.84%)	1.25(1.51%)	21.5(25.84%)	-1.68(-79.83%)	-294.98(-355.42%)	-292.32(-351.33%)	-20.87(-9923%)
high-middle	-89.77	-115.1	25.33	427.11(475.77%)	389.65(338.52%)	36.07(142.4%)	77.16(85.95%)	98.9(85.92%)	4.9(19.34%)	-594.04(-661.72%)	-603.65(-524.44%)	-15.64(-61.73%)
middle	532.92	468.36	64.56	544.66(102.2%)	469.36(100.21%)	70.72(109.54%)	398.05(74.69%)	327.17(69.85%)	54.11(83.8%)	-409.79(-76.9%)	-328.17(-70.07%)	-60.26(-93.34%)
low-middle	510.17	443.39	66.79	400.81(78.56%)	361.78(81.59%)	35.27(52.81%)	221.36(43.39%)	172.68(38.95%)	21.92(32.83%)	-112(-21.95%)	-91.07(-20.54%)	9.59(14.36%)
low	114.43	96.97	17.46	114.43(99.8%)	99.48(102.59%)	13.78(78.95%)	59.54(52.03%)	46.38(47.83%)	8.06(46.15%)	-59.31(-51.83%)	-48.89(-50.42%)	-4.38(-25.09%)

Abbreviations: OESA=occupational exposure to sulfuric acid asbestos.

Supplementary Table 3 Changes in deaths of laryngeal cancer atrributable to OEA according to decomposition analysis from 1990 to 2021 in global and SDI regional level.

	Overall			Population growth			Aging			Epidemiological changes		
	both	male	female	both	male	female	both	male	female	both	male	female
Global	597.15	536.18	60.97	1746.83(292.53%)	1667.94(311.08%)	81.97(134.43%)	1046.2(175.2%)	1199.93(223.79%)	47.02(77.11%)	-2195.88(-367.73%)	-2331.69(-434.87%)	-68.01(-111.54%)
SDI regions												
high	46.4	31.79	14.61	840.89(1812.37%)	815.96(2566.71%)	35.07(240.11%)	225.85(486.77%)	439.99(1384.06%)	5.39(36.9%)	-1020.34(-2199.14%)	-1224.16(-3850.77%)	-25.86(-177.01%)
high-middle	-44.83	-57.16	12.33	523.88(1168.66%)	505.53(-884.47%)	22.75(184.49%)	216.03(481.93%)	304.68(533.07%)	8.42(68.33%)	-784.74(-1750.59%)	-867.37(-1517.54%)	-18.84(-152.82%)
middle	297.98	274.68	23.3	195.71(65.68%)	176.03(64.09%)	18.46(79.21%)	202.46(67.95%)	183.47(66.8%)	20.35(87.32%)	-100.19(-33.62%)	-84.83(-30.88%)	-15.5(-66.53%)
low-middle	242.68	233.17	9.51	121.19(49.94%)	115.55(49.56%)	4.57(48.09%)	88.59(36.5%)	72.17(30.95%)	4.19(44.04%)	32.9(13.56%)	45.44(19.49%)	0.75(7.87%)
low	53.49	52.35	1.15	32.78(61.28%)	32.01(61.15%)	0.48(41.46%)	17.6(32.89%)	15.03(28.72%)	0.3(25.9%)	3.12(5.82%)	5.3(10.13%)	0.37(32.65%)

Abbreviations: OEA=occupational exposure to asbestos.

Supplementary Table 4 Top fifteen countries and territories with effective difference in ASDR in 2021 for laryngeal cancer atrributable to OESA and OEA according to frontier analysis.

	OESA			OEA		
	**Effective difference in ASDR, per 100,000**	**SDI level**	**Which category of SDI region**	**Effective difference in ASDR, per 100,000**	**SDI level**	**Which category of SDI region**
Countries and territories						
1st	Cuba (0.18)	0.6687	middle	Monaco (0.36)	0.9083	high
2nd	Pakistan (0.14)	0.5040	low-middle	Lesotho (0.29)	0.5104	low-middle
3rd	Seychelles (0.11)	0.7302	high-middle	Croatia (0.21)	0.7983	high-middle
4th	Saint Vincent and the Grenadines (0.09)	0.6372	middle	France (0.18)	0.8384	high
5th	Georgia (0.09)	0.7325	high-middle	Namibia (0.18)	0.6176	low-middle
6th	Uruguay (0.08)	0.7193	high-middle	Eswatini (0.15)	0.5855	low-middle
7th	Haiti (0.08)	0.4483	low	Italy (0.15)	0.8058	high-middle
8th	Romania (0.08)	0.7685	high-middle	Saint Kitts and Nevis (0.14)	0.7550	high-middle
9th	Montenegro (0.07)	0.7958	high-middle	Belgium (0.13)	0.8537	high
10th	Bangladesh (0.07)	0.4924	low-middle	Greenland (0.12)	0.8262	high
11th	Hungary (0.07)	0.7908	high-middle	Turkey (0.12)	0.7127	high-middle
12th	Dominica (0.07)	0.7470	high-middle	United Kingdom (0.12)	0.8590	high
13th	Bulgaria (0.07)	0.7682	high-middle	Poland (0.11)	0.8120	high
14th	Brazil (0.07)	0.6530	middle	Denmark (0.11)	0.8964	high
15th	India(0.07)	0.5754	low-middle	South Africa (0.11)	0.6796	middle

Abbreviations: ASDR = age standardized death rate; OESA=occupational exposure to sulfuric acid asbestos ; OEA=occupational exposure to asbestos.

Supplementary Table 5 The future number of deaths and ASDR of laryngeal cancer atrributable to OESA and OEA from 2022 to 2040 by BAPC model.

	**2022**		**2040**	
	**Number of deaths, (95% UI)**	**ASDR per 100 000 (95% UI)**	**Number of deaths, (95% UI)**	**ASDR per 100 000 (95% UI)**
**OESA**				
Global	3679.08(3137.17, 4225.18)	0.06(0.05, 0.07)	4810.90 (1628.05, 8010.74)	0.06(0.02, 0.10)
males	3250.78(2846.09, 3656.57)	0.12(0.11, 0.12)	4205.52(1539.47, 6877.95)	0.11(0.04, 0.17)
females	428.30(291.08, 568.60)	0.01(0.01, 0.02)	605.38(88.58, 1132.79)	0.01(0.00, 0.02)
**OEA**				
Global	3441.53(2950.53, 3937.46)	0.06(0.05, 0.07)	3648.48(1016.75, 6322.42)	0.04(0.01, 0.07)
males	3254.56(2848.76, 3662.29)	0.14(0.13, 0.14)	3395.39(1016.75, 5779.16)	0.08(0.03, 0.12)
females	186.98(101.77, 275.17)	0.01(0.01, 0.01)	253.10(0, 543.25)	0.01(0, 0.01)

Abbreviations: ASDR = age standardized death rate; OESA=occupational exposure to sulfuric acid; OEA=occupational exposure to asbestos; UI = uncertainty interval; BAPC=Bayesian age-period-cohort.

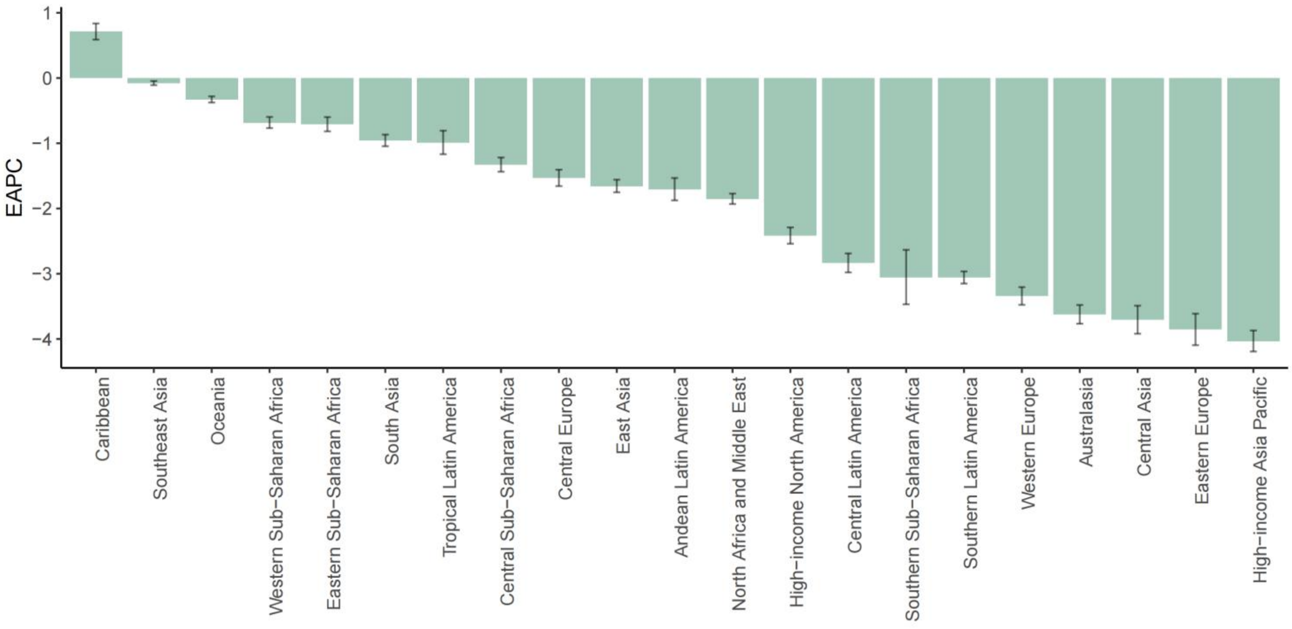

Supplementary Figure 1. The EAPC of laryngeal cancer atrributable to OESA from 1990 to 2021 among 21 regions. EAPC: estimated annual percentage change; OESA: occupational exposure to sulfuric acid.

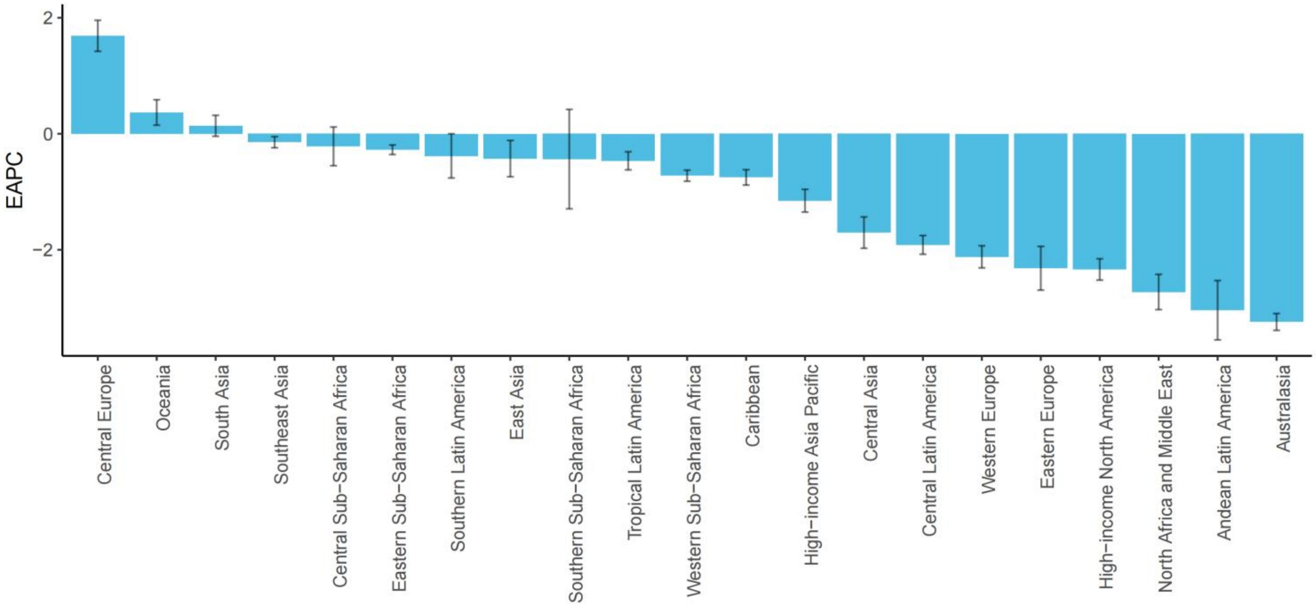

Supplementary Figure 2. The EAPC of laryngeal cancer atrributable to OEA from 1990 to 2021 among 21 regions. EAPC: estimated annual percentage change; OEA: occupational exposure to asbestos.

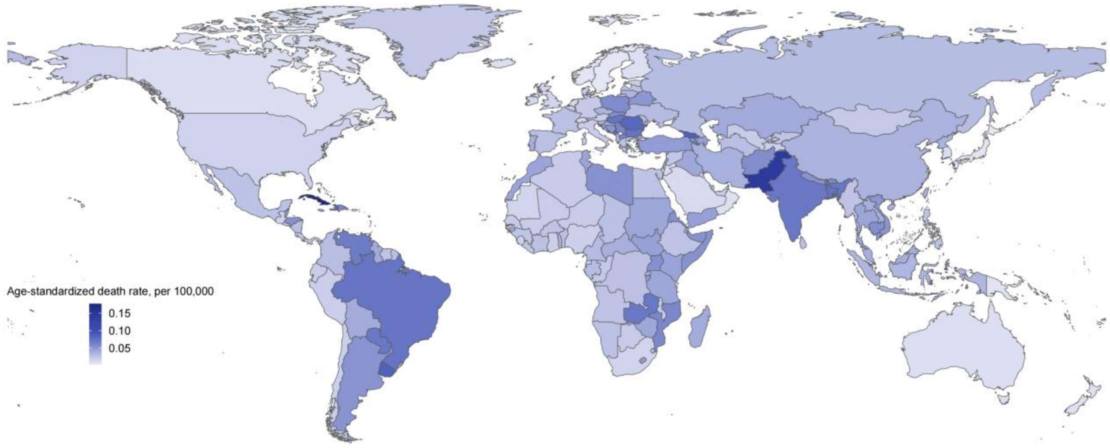

Supplementary Figure 3. The Age-standardized death rates of laryngeal cancer atrributable to OESA per 1000,000 among 204 countries and territories in 2021. OESA: occupational exposure to sulfuric acid.

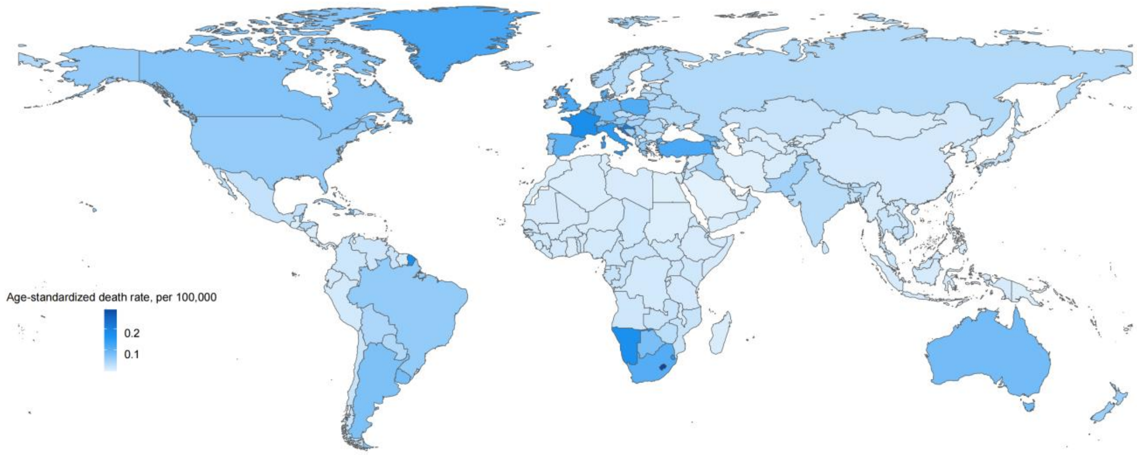

Supplementary Figure 4. The Age-standardized death rates of laryngeal cancer atrributable to OEA per 1000,000 among 204 countries and territories in 2021. OEA: occupational exposure to asbestos.

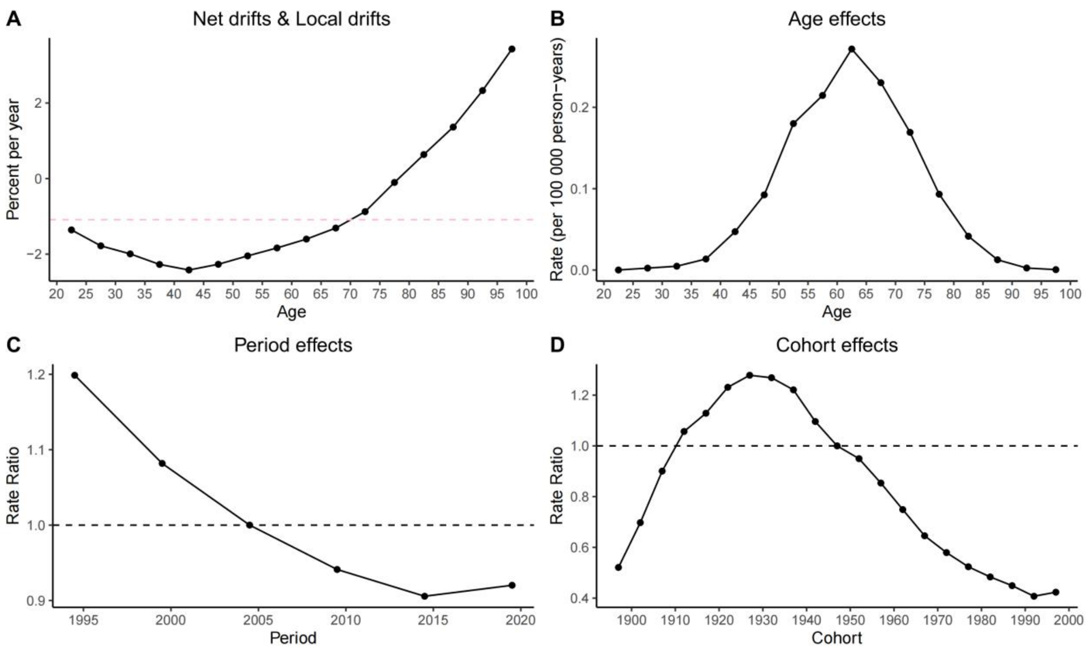

Supplementary Figure 5. The Age-standardized death rate relative risks of laryngeal cancer atrributable to OESA due to age, period and cohort effects globally. (A) The net drifts and local drifts. (B) The age effect. (C) The period effect. (D) Birth cohort effect. OESA: occupational exposure to sulfuric acid.

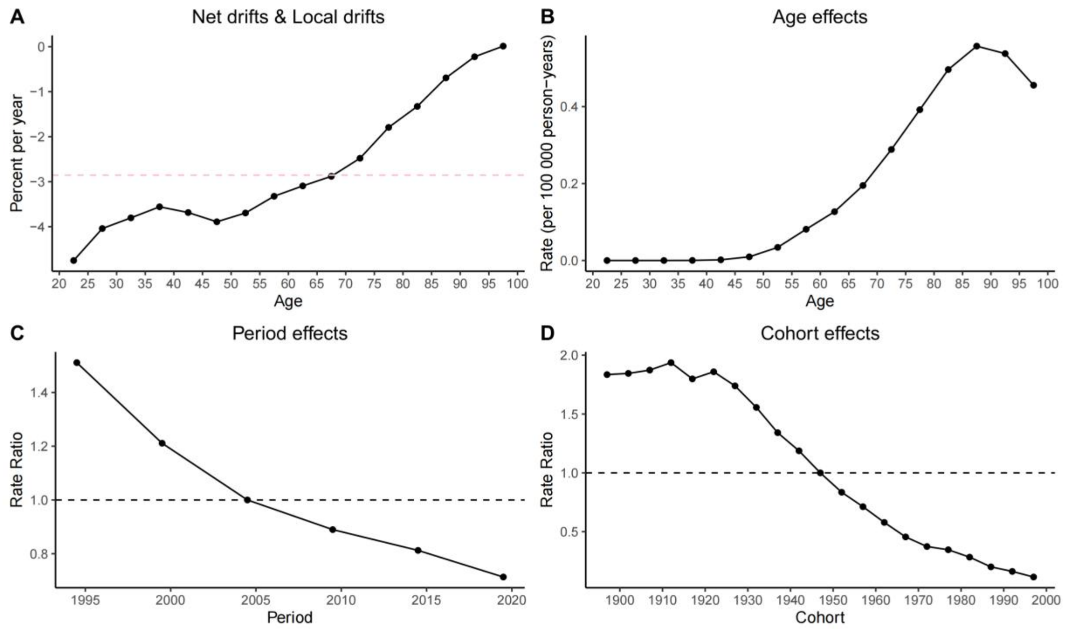

Supplementary Figure 6. The Age-standardized death rate relative risks of laryngeal cancer atrributable to OEA due to age, period and cohort effects globally. (A) The net drifts and local drifts. (B) The age effect. (C) The period effect. (D) Birth cohort effect. OEA: occupational exposure to asbestos.
