# Supplementary figures and images for: Global, regional and national mortality burden of laryngeal cancer attributable to occupational exposure to sulfuric acid and asbestos: 1990–2021 and projections to 2040
Source: Front Public Health. 2025 Jul 18;13:1602789. doi: 10.3389/fpubh.2025.1602789 (PMC12315700; doi:10.3389/fpubh.2025.1602789)

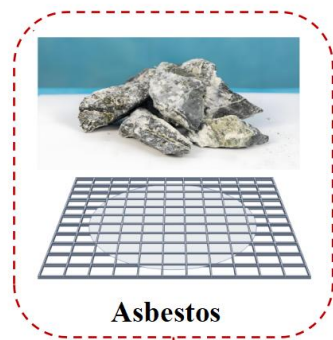

Asbestos

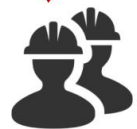

Occupational exposure

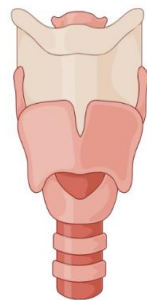

Laryngeal cancer

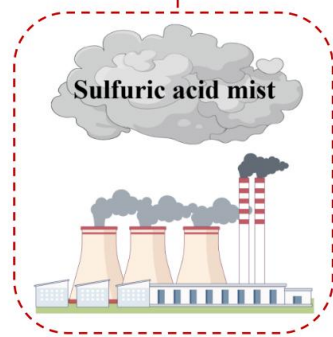

Sulfuric acid mist

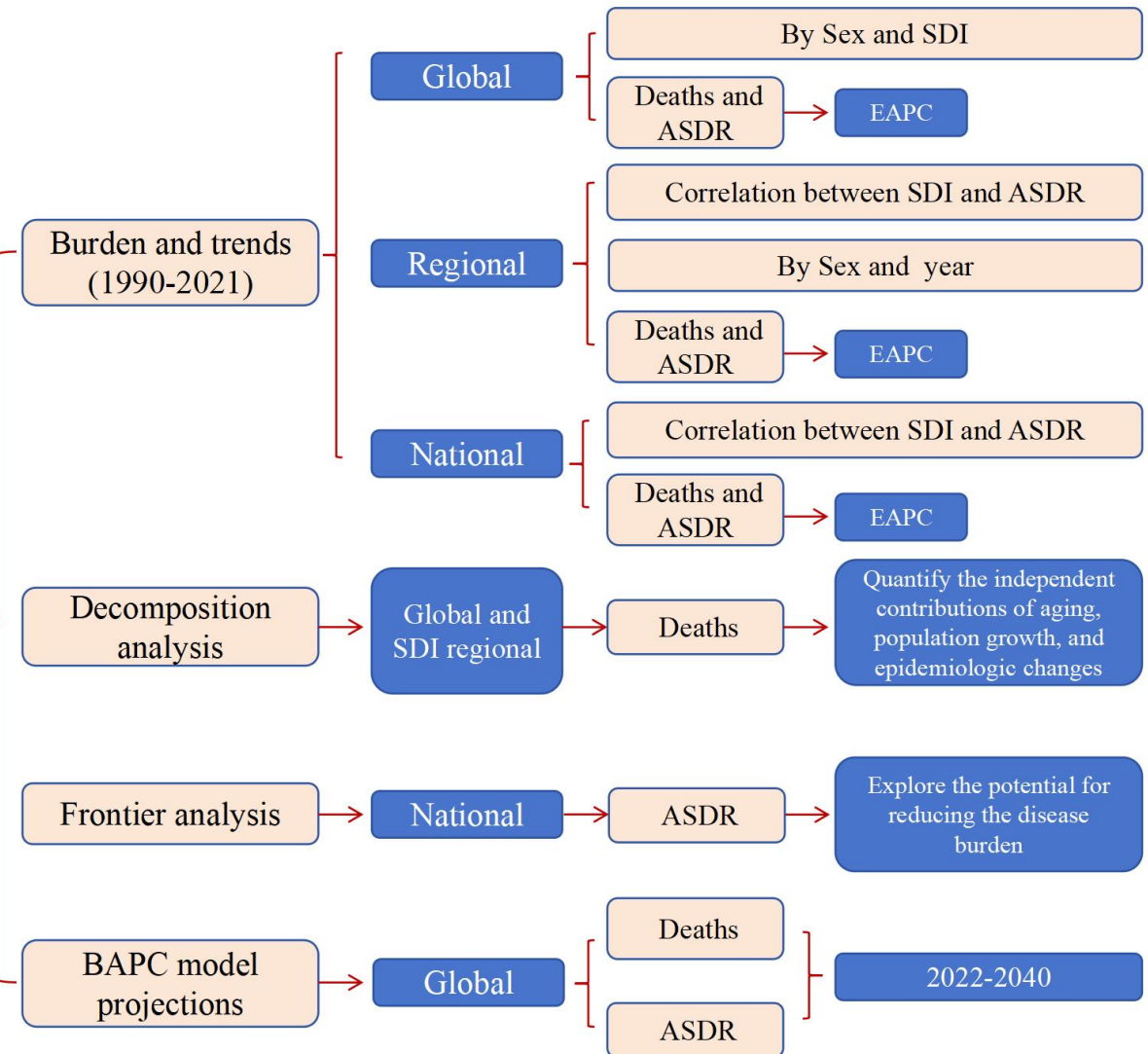

Supplement: Supplementary file 3 [file Data_Sheet_3.pdf]
